# The ring vaccination trial: a novel cluster randomised controlled trial design to evaluate vaccine efficacy and effectiveness during outbreaks, with special reference to Ebola

**DOI:** 10.1136/bmj.h3740

**Published:** 2015-07-27

**Authors:** Ebola ça suffit ring vaccination trial consortium

## Abstract

A World Health Organization expert meeting on Ebola vaccines proposed urgent safety and efficacy studies in response to the outbreak in West Africa. One approach to communicable disease control is ring vaccination of individuals at high risk of infection due to their social or geographical connection to a known case. This paper describes the protocol for a novel cluster randomised controlled trial design which uses ring vaccination.

In the Ebola ça suffit ring vaccination trial, rings are randomised 1:1 to (*a*) immediate vaccination of eligible adults with single dose vaccination or (*b*) vaccination delayed by 21 days. Vaccine efficacy against disease is assessed in participants over equivalent periods from the day of randomisation. Secondary objectives include vaccine effectiveness at the level of the ring, and incidence of serious adverse events.

Ring vaccination trials are adaptive, can be run until disease elimination, allow interim analysis, and can go dormant during inter-epidemic periods.

**Key points**

- Evaluating the efficacy of novel vaccines and therapeutics in epidemics is challenging, with situations evolving rapidly and ethical concerns about research during public health emergencies
- Ring vaccination around cases was used successfully in the smallpox eradication programme
- A novel cluster randomised control trial of immediate versus 21 day delayed ring vaccination against Ebola is underway in Guinea
- A ring vaccination trial tracks the epidemic, recruiting individuals at raised risk of infection due to their connection to a case: this design may both contribute to transmission interruption and have a higher power to detect vaccine efficacy than other study designs

Evaluating vaccine efficacy during outbreaks can be challenging due to the timescales involved, ethical concerns around research methods, and field operational challenges such as cold chain management and effective communication with those affected. Furthermore, to have adequate statistical power to detect a vaccine effect, a sufficient number of events must be observed. These challenges have again come to international attention due to the devastating epidemic of Ebola virus disease (EVD) in West Africa,^1^ where weak infrastructure for health and development exacerbate the difficulties inherent in communicable disease control and related interventional research.

An approach to increasing vaccine study power is to recruit those at highest risk of infection. A trial might thus recruit individuals who are socially or geographically connected to a case and therefore at increased risk of infection and developing disease within a few weeks. When implemented as a targeted programmatic public health measure, such an approach is described as “ring vaccination.”

A surveillance-containment strategy using ring vaccination was central to smallpox eradication in the 1970s. This contributed to the interruption of transmission in Africa, South America, and Asia.^2^ Ring vaccination with an efficacious vaccine might similarly help to control other communicable diseases by creating a buffer of immune people around each new case, thereby preventing further spread of the infection. Simulation studies suggest ring vaccination can contain outbreaks of infectious diseases with relatively low reproduction numbers (R0),^3^ such as EVD, for which R0 has been estimated at between 1 and 3.^4^ ^5^ Some studies note that effective contact tracing, case isolation, and quarantine or monitoring of cases can have an effect equivalent to ring vaccination.^3^ ^6^ A ring vaccination trial therefore tests both the vaccine and the approach.

In this paper, we describe the design considerations behind the protocol for a ring vaccination trial, a novel cluster randomised controlled trial to evaluate vaccines against EVD in Guinea, West Africa.

## The Ebola ça suffit randomised ring vaccination trial

In the Ebola ça suffit (“Ebola, that’s enough”) ring vaccination trial, a person newly diagnosed with EVD becomes the index case around whom an epidemiologically defined ring is formed. This ring is then randomised to either immediate vaccination (intervention) or delayed vaccination (control) in a 1:1 ratio on an open label basis. The incidence of disease is compared between the two arms over equivalent time periods measured from the time of randomisation of each ring. Comparing the hazard ratio in those enrolled in the study allows estimation of vaccine efficacy, while overall vaccine effectiveness can be estimated by comparing incidence across all members of the rings, including those not eligible for vaccination in the study.

## Intervention and control

The trial tests the recombinant vesicular stomatitis virus Ebola vaccine (rVSV-ZEBOV), which was developed by the Public Health Agency of Canada, and licensed to NewLink Genetics and Merck. rVSV-ZEBOV was selected based on its safety profile, induction of potentially protective immune responses, including neutralising antibodies, and availability of vaccine doses.

In a ring vaccination trial, the control arm could be a placebo or a vaccine against a disease not under study. This was deemed unacceptable in Guinea because of national and international concerns about leaving vulnerable individuals unprotected against EVD when a potentially effective vaccine was available.^7^ An assessment of the epidemiology of EVD in Guinea was done, which suggested that a 21 day delay, the incubation period in which 95% of EVD cases arise,^5^ for the control arm could be sufficient to determine efficacy, while meeting the requirement to minimise study participants’ time without vaccination. In Guinea the trial is open label, but in settings with fewer operational challenges a ring vaccination trial of immediate versus delayed vaccination could include blinding of allocation by using additional visits to each study arm for the administration and follow-up of a placebo or non-study vaccine. In the Ebola ça suffit ring vaccination trial, both the immediate and delayed vaccination arms receive equivalent infection prevention and control advice at enrolment. This includes informing study participants that the vaccine may not offer protection, so they should not take risks with Ebola exposure, and that the vaccine may not prevent Ebola in people already infected.

## Design and implementation

The recruitment of ring vaccination trial study participants is driven by the detection of new cases. After notification of a laboratory confirmed EVD case in Basse-Guinea, the designated trial area, a contact list is drawn up by study field teams using the World Health Organization (WHO) contact tracing record.^8^ The newly diagnosed EVD case becomes the index case around which an epidemiologically defined ring is formed comprising the person’s contacts and contacts of contacts who may also be at raised risk of EVD. This second tier of people is of critical importance to the trial and the public health intervention. The timing of exposure and intervention means that contacts may already be incubating the virus at the time of vaccination. Secondary cases in these individuals may not be averted unless there is a strong post-exposure prophylactic effect from the vaccine. However, post-exposure prophylactic effect is not an objective of the Guinea ring vaccination trial. The trial is instead premised on immediate vaccination providing rapid pre-exposure prophylaxis in averting later case generations when compared with delayed vaccination. This is illustrated in figure 1. 

**Figure fig1:**
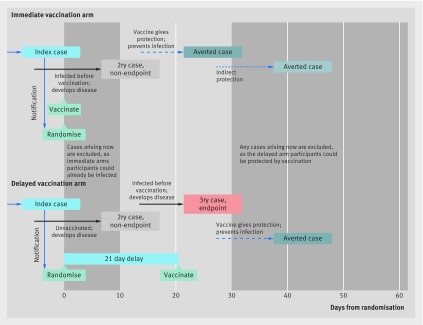
**Fig 1** Development of cases arising in two hypothetical, simplified, example trial rings, which are identical but for receipt of immediate or delayed (by 21 days) vaccination. Case boxes indicate the presence of symptoms and infectiousness. Arrows indicate disease incubation periods after infection. Because disease onset is detected rather than the point of infection, the shaded areas show periods in which any cases arising would be excluded from an analysis of vaccine efficacy against infection. This example shows a window which assesses the vaccine’s ability to offer protection from the day of administration. Three cases arise during the trial; one contributes to the analysis

Contacts are defined as individuals who, within the previous 21 days, lived in the same household as the symptomatic patient, were visited by the symptomatic patient, or were in close physical contact with the patient’s body or body fluids, linen, or clothes.^8^ Contacts of contacts are defined as the neighbours or extended family members to nearest geographic boundary in which the local contacts of the index case reside, plus household members of any high risk contacts who do not live in the same locality as the case (further details are in the accompanying protocol). The ring is not necessarily a contiguous geographic area but captures a social network of individuals and locations that may include dwellings or workplaces further afield, where the index patient spent time while symptomatic, or the households of individuals who had contact with the patient during the illness or after his or her death.

Before vaccination related activities are initiated, local social mobilisation experts visit the residents of the main ring site around the index patient’s residence, seek their consent for the trial team to approach them, through community leaders and representatives where applicable, and explain the trial’s objectives and the implications of potential participation. If consent is granted for the ring site to be included in the vaccine trial, a social mobiliser works with trial staff to define the ring, drawing up a list of all contacts and contacts of contacts in the ring, regardless of their eligibility for vaccination in the trial. The process is repeated for satellite sites of the same epidemiological ring. Randomisation is done by telephone from the main ring site after the ring is defined and potential participants determined, stratified by location (urban or rural) and number of ring members (≤20 or >20). The allocation sequence was generated by an independent statistician using randomly varying block lengths and is held on the electronic data management system with no access for staff involved in study recruitment.

Once the ring is defined and randomised, informed consent is obtained from willing and eligible members of the ring. Allocation is not revealed to participants until after informed consent. A list is also established of all contacts and contacts of contacts who are either non-eligible or eligible but not enrolled in the trial. Reasons for non-enrolment are recorded. After enrolment, teams visit to administer vaccination as per the randomised schedule.

An important concern is that the Ebola ça suffit trial must not detract from patient care or communicable disease control. To avoid the trial placing additional demands on outbreak control teams, there are dedicated study field teams which support and work closely with the Ebola contact tracing and surveillance teams. Potential cases identified in the course of the study are assessed by a study physician using a national protocol and referred to Ebola treatment units for diagnosis and clinical management as appropriate.

## Primary and secondary outcomes

New cases of EVD in the ring are ascertained through active and passive surveillance using visits to the ring members, telephone notification from ring representatives, and case detection through the national EVD surveillance system. Contacts are followed up in accordance with usual surveillance practices. Confirmed cases arising in enrolled ring members during the relevant ascertainment window are included as primary outcomes in the main analysis of vaccine efficacy. Suspected or probable EVD and death from confirmed EVD are included as secondary outcomes. Confirmed EVD cases in non-enrolled ring members contribute to secondary analyses of vaccine effectiveness, including analyses estimating indirect effects of vaccination, as described in the box and the section on statistical analysis. Data on serious adverse events will be collected throughout the trial.

**Synopsis of Ebola ça suffit randomised controlled trial: a ring vaccination trial to evaluate the safety and efficacy of an Ebola vaccine in Guinea, West Africa (PACTR201503001057193)**

**Setting**

Basse Guinea, the area of the country where most of the EVD cases are being reported by March 2015. It includes five prefectures: Conakry, Coyah, Kindia, Forécariah, Dubreka.

**Primary objective**

To assess vaccine efficacy against confirmed Ebola virus disease (EVD) by performing a clinical trial comparing immediate versus delayed ring vaccination.

**Secondary objectives**

- To assess vaccine effectiveness (indirect and overall) in preventing confirmed EVD, using both direct calculation and transmission dynamic modelling
- To assess vaccine efficacy against death from confirmed EVD
- To assess vaccine efficacy against probable and suspected EVD
- To evaluate vaccine safety by assessing serious adverse events.

**Randomised comparison**

Immediate vaccination of rings with a single dose is compared with delayed vaccination of rings with single dose, with a delay of 21 days.

**Definition of rings and inclusion criteria**

Laboratory confirmed EVD cases trigger visits for ring definition. Based on contact tracing and places of residence of the case, an epidemiologically defined sociogeographical ring is identified. Individuals aged ≥18 years (excluding pregnant or breastfeeding women and others meeting exclusion criteria) who live in the defined vaccination ring are offered vaccination.

**Outcomes**

Confirmed EVD is the primary outcome. Secondary outcomes include suspected and probable cases and serious adverse events (see WHO details of contact tracing during an EVD outbreak^8^ for case definitions).

**Study assessments and follow-up**

The study team enumerates the residents of the ring, seeks their informed consent, assesses eligibility, and vaccinates them if they are willing and eligible. Follow-up visits are conducted at days 3, 14, 21, 42, 63, and 84 after vaccination. The study team inquires about any symptoms or signs to ascertain the occurrence of serious adverse events or EVD and any other changes in health status of volunteers since previous visit.

**Sample size**

Approximately 190 rings (95 per arm) of size 50 are required to have 90% power to reject the null hypothesis. The final sample size achieved will depend on the number of new index cases accumulating during the study period. Interim analysis is planned using an alpha spending strategy that may allow the trial to conclude early if compelling evidence of a significant protective vaccine effect is observed (see main text).

**Risk of bias**

Random allocation of vaccination rings is done remotely, by a member of the study team not involved in the definition of rings. The study is open label, so blinding of participants and field teams is not possible. Informed consent and eligibility are done after randomisation of each ring, with participants advised of their allocation after informed consent. Communicable disease control measures other than ring vaccination are identical in the two groups.

If ring members develop confirmed EVD they are also assessed as potential new index cases. If at least 60% of the second case’s contacts and contacts of contacts are outside the first case’s ring then a second ring that includes all individuals not already included in the trial is defined and randomised. The 60% threshold was based on field operational consideration, with the intention to balance the possible opportunity cost of not recruiting another distant ring with the evidential gain from further investigation of a ring around a secondary case.

The box and figure 2 summarise the design and analysis plan of the trial. Full details of the inclusion and exclusion criteria and study conduct are in the study protocol (available in supplementary data on bmj.com). An annotated SPIRIT checklist is also provided in the supplementary data.^9^

**Figure fig2:**
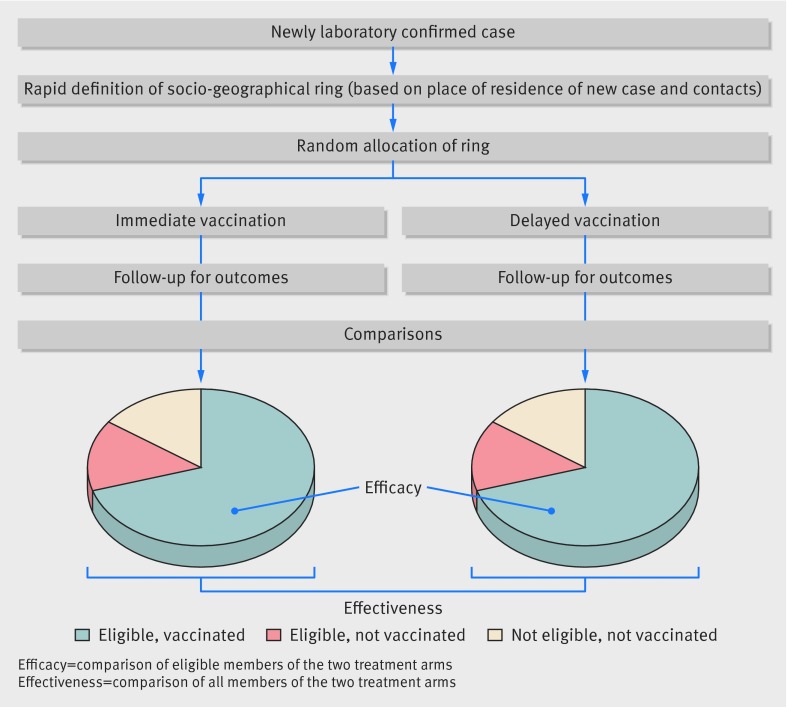
**Fig 2** Schematic presentation of the design of a ring vaccination trial during an outbreak of an infectious disease

## Ethical and regulatory approvals

Approval to perform the trial was obtained from the Guinean national ethics committee (Comite National d’Ethique pour la Recherche en Sante), the Ebola research commission (Commission Recherche Ébola en Guinée), the WHO Ethical Research Committee, and the Regional Committees for Medical and Health Research Ethics (REC) in Norway. Regulatory approval was obtained from the national medicines regulatory agency in Guinea (Direction Nationale de la Pharmacie et du Laboratoire), with regulatory review supported by Health Canada. The trial is registered with the Pan African Clinical Trials Registry (PACTR201503001057193).

## Statistical analysis of ring vaccination trials

As vaccination rings can be viewed as clusters and because randomisation occurs at the level of the ring, the extensive literature on the design, analysis, and reporting of cluster randomised trials can be readily adopted.^10^

The primary analysis in a ring vaccination trial estimates vaccine efficacy against disease. Vaccine efficacy is defined as *VE*=1−è, where è=ë_1_/ë_0_ is the hazard ratio of ë_1_ (the hazard of disease for eligible and vaccinated individuals in a ring who receive immediate vaccination) and ë_0_ (hazard of disease for eligible individuals in a ring who receive delayed vaccination before individuals in the ring are vaccinated). To capture events that can be used for the estimation of vaccine efficacy, the analysis period is shifted in time. This delay incorporates time for vaccinated individuals to develop protective immunity and for disease incubation, as symptom onset times are observed in the trial rather than the infection times.

The hypothesis test for the primary outcome is *H_0_: VE*=0 versus *H_a_: VE≠*0*.* The hazard ratio can be estimated using a Cox proportional hazards regression model. For clustered time to event data, available methods include random effects models, also known as frailty models, and stratified proportional hazards models.^11^ In the Guinea Ebola ça suffit trial, we will include a frailty value for each ring.^12^ If the number of rings is small, imbalances between the comparison groups are likely to occur by chance alone. Imbalances in important variables can be adjusted for in the analysis by incorporating measured confounders as additional covariates.

To estimate overall vaccine effectiveness, a ring vaccination trial with delayed vaccination can compare the incidence of disease between rings randomised to immediate or delayed vaccination by including events among unvaccinated individuals in all rings.^13^ In addition, it is possible to estimate direct vaccination and indirect vaccination effects. This includes the degree to which unvaccinated people are protected in rings at different levels of vaccine coverage.

## Study power and sample size

As with any cluster randomised trial, the sample size must be inflated for the effect of clustering within rings as the members of a ring share a common exposure to the index case and are not statistically independent.^14^ ^15^ The design effect, the amount by which the sample size must be inflated, depends both on the degree of correlation within rings (that is, the intraclass correlation coefficient) and the size of the rings. If the design effect is high, power will mainly be determined by the number of rings rather than the total number of individuals enrolled.^14^ ^15^ If ring size is expected to vary widely, it may be advisable to further inflate the design effect to account for a potential reduction in study precision.^16^

Sample size calculations may be based on the total number of events that need to be observed to yield a particular level of power to detect a given vaccine efficacy, or they can be based on the total number of rings that must be followed, assuming a particular event rate and ring size. To estimate the design effect for the trial, it is necessary to assume a particular intraclass correlation coefficient. Such a value can be approximated from available pilot data, surveillance records, or the literature.

For the Ebola ça suffit ring vaccination trial, if true vaccine efficacy is 70%, and intraclass correlation coefficient is 0.05 (based in part on analysis of unpublished data summarising transmission chains in Guinea, including occurrence of superspreading events) approximately 190 rings (95 per arm) of size 50 are required to have 90% power to reject the null hypothesis. A fixed ring size was used in power calculations as there was limited data on variability in cluster size in the early design stages, and the impact of cluster size variability is less critical than estimated event rate and intraclass correlation coefficient.

The trial design lends itself naturally to adaptive approaches to statistical analysis, since estimation and hypothesis testing can be done as rings accumulate.^17^ For the Ebola ça suffit trial, interim analysis will be performed using an alpha spending strategy with truncated O’Brien-Fleming boundaries and a first review likely around 100 rings.^18^ Interim analysis will allow the trial to be stopped early if there is compelling evidence of vaccine efficacy, which may allow the vaccine to be more quickly deployed outside of a study.

## Discussion

The Ebola ça suffit ring vaccination trial design proposed here was developed in response to the urgent need to evaluate experimental vaccines against Ebola virus disease (EVD) and began in April 2015.

The ring vaccination trial design combines approaches from clinical trial methodology, infectious disease epidemiology, and applied public health into a pragmatically informed efficacy and effectiveness trial that can be implemented in a resource-poor country during an epidemic. The design allows evaluation of the efficacy of the vaccine at the individual level and of the effectiveness of ring vaccination as a containment strategy. A ring vaccination trial therefore both implements and evaluates a public health intervention.

A practical advantage of the ring vaccination trial design is that all eligible participants within the ring can be vaccinated and followed up around the same time in the same location, at their place of residence. In addition, the design is less affected by low incidence than a standard parallel group design, as it is always being conducted in small pockets of high risk individuals. Variations on this trial design may thus in future be used to evaluate vaccines against rare diseases, such as meningococcal disease, or to evaluate vaccines that become available towards the end of an outbreak. Indeed ring vaccination trials need not be limited to vaccines but could evaluate, for example, group health education or chemoprophylaxis given in response to infectious disease cases.

Because of extensive field operational challenges—including community resistance, difficulty reaching remote field sites, and vaccine transportation at −80°C—the Ebola ça suffit trial forgoes two of the routine practices of randomised controlled trials. The first is that there are no placebo vaccination visits for double blinding. To reduce the risk of bias arising from behaviour changes that might follow vaccination, participants are informed that it is not known if the vaccine works and that they must still take steps to avoid infection. The second is that rings are randomly allocated before individual informed consent is obtained. Although the consent team are aware of allocation, making this de facto unconcealed, participants are told of their vaccination schedule only at the end of the informed consent process. Monitoring of recruitment to date has not indicated differences between study arms, though selection bias cannot be excluded.

The ring vaccination trial design shares the limitations of cluster randomised controlled trials.^14^ ^19^ The need to increase sample size due to cluster randomisation can, however, be balanced by the substantially higher event rate in ring vaccination trial participants compared with an individually randomised trial in the general population of an affected area, which may result in a smaller overall sample size. The biases that are particularly relevant to cluster randomised controlled trials also apply to ring vaccination trials, including imbalances in important variables at the level of the ring.^19^ The Ebola ça suffit trial initiates new rings linked to secondary cases in study rings in order to maximise the number of rings recruited; this omits buffer zones used to reduce inter-cluster contamination.^15^ Potential confounding variables—including participation rates, index case characteristics such as days to isolation, and comparability of rings, ring populations, and drop-out rates—can be measured and adjusted for. Intensive follow-up regimens and use of national case surveillance can reduce the likelihood of differential ascertainment of endpoints.

A ring vaccination trial using delayed vaccination as the control arm addresses one of the ethical concerns of placebo controlled trials, which are seen to arbitrarily deny some participants access to potentially effective interventions.^7^ Such a design is more complex to analyse than a placebo controlled study. Crucially, it is important to clarify the equivalent time periods for which the comparison is made. The Ebola ça suffit trial’s primary analysis uses a fixed delay to account for the incubation period and the time for development of immunity after vaccination, both of which are expected to vary across individuals. The impact will be some misclassification of trial events resulting in an estimate of vaccine efficacy that is biased towards the null. Further details are described in the statistical analysis plan and a forthcoming publication.

As noted, a ring vaccination trial tests both the vaccine and the vaccination strategy. It is possible that in a ring vaccination trial, an efficacious vaccine may not be shown to be so, either because the ring vaccination delivery strategy or the ring vaccination trial design is unsuitable for the vaccine. This may be due to the concurrent non-vaccine control measures, or to issues with the timing of vaccine delivery and immunity onset relative to exposure.

In conclusion, we propose a novel design to estimate efficacy and effectiveness of experimental vaccines during outbreaks, and have implemented this design to test a vaccine against EVD in Guinea. Although the number of new EVD cases has declined in Guinea in recent months, it is possible that the epidemic will continue for some time at a lower intensity. We hope that the vaccine tested in the Ebola ça suffit trial will contribute to ending the epidemic. Should efficacy not be demonstrated in the current outbreak, the adaptive nature of the ring vaccination trial design would make it plausible to issue a preliminary report after cessation of transmission and to reactivate or initiate a modified version of the trial when the next Ebola zoonotic transmission event occurs.^20^

## Notes

The authors (and members of the Ebola ça suffit ring vaccination trial consortium, who contributed to this article) are (in alphabetic order): Anton Camacho, Miles W Carroll, Natalie E Dean, Moussa Doumbia, W John Edmunds, Matthias Egger, Godwin Enwere, Yper Hall, Ana Maria Henao-Restrepo, Stefanie Hossmann, Sakoba Keita, Mandy Kader Kondé, Ira M Longini, Sema Mandal, Gunnstein Norheim, Ximena Riveros, John-Arne Røttingen, Sven Trelle, Andrea S Vicari, Sara V Watle, and Conall H Watson.

We thank Professor D A Henderson for helpful discussions regarding the use of surveillance and containment to eradicate smallpox and its application to the design of the ring vaccination trial. We also thank Bertrand Draguez, Rebecca Grais, Marie Paule Kieny, Myron Levine, Peter Smith, and Aboubacar Soumah for their advice and guidance, and to colleagues at CTU Bern for support with data management.

The development of the design of the Ebola ça suffit trial was supported by the Guinean authorities, WHO, Médecins Sans Frontières (MSF) Belgium and Epicentre, the Norwegian Institute of Public Health, the University of Florida, the London School of Hygiene and Tropical Medicine, the University of Bern, the University of Maryland, the Center for Vaccine Development Mali, Public Health England (Colindale and Porton Down), Public Health Agency of Canada, NewLink/Merck, and European Mobile Laboratory Guinea.

Contributors: All authors critically reviewed the manuscript for content. AC provided advice on trial design; extracted, cleaned, and analysed epidemiology data on Ebola in Guinea to inform sample size calculations; and helped draft the manuscript. MWC provided advice on trial design and protocol, supported and designed the process for documentation of laboratory confirmation of Ebola cases, and contributed to the drafting of the manuscript. NED provided advice on trial design, contributed to the protocol development, designed the statistical analysis plan, and helped write the first draft the manuscript. MD contributed to the protocol and provided inputs on operational issues regarding trial design and implementing the trial, and helped draft the manuscript. WJE conceived and designed the trial, analysed epidemiology data on Ebola in Guinea to inform sample size calculations, and helped draft the manuscript. ME conceived and designed the trial, prepared the first draft of the protocol, contributed to the statistical analysis plan, and prepared the first draft of and revised the manuscript. GE contributed to the design of the trial, contributed to the protocol development, designed trial safety procedures, implemented the trial, and helped draft the manuscript. YH provided inputs on trial design and protocol, supported process for documentation of laboratory confirmation of cases, and helped draft the manuscript. AMHR conceived and designed the trial, coordinated all aspects of the design and implementation of the trial, contributed to the protocol, contributed to the design of data collection systems, helped draft the manuscript, revised the manuscript, and is guarantor. SH contributed to the protocol, designed data collection systems, implemented data management, and helped draft the manuscript. SK contributed to the protocol, provided advice on operational issues regarding trial design, implemented the trial, and helped draft the manuscript. MKK contributed to the design of the trial, provided advice on operational issues regarding trial design and implementing the trial, and helped draft the manuscript. IML conceived and designed the trial, designed the statistical analysis plan, helped prepare the first draft the manuscript and revised the manuscript. SM contributed to the design of the trial, revised the protocol, contributed to the design of data collection systems, and helped draft the manuscript. GN conceived and designed the trial, prepared the first draft of the protocol, contributed to ethical and regulatory approval and community engagement planning, and helped draft the manuscript. XR contributed to the design of the trial, revised the protocol, coordinated implementation and logistics, and helped draft the manuscript. JAR contributed to the design of the trial and to the protocol and helped draft the manuscript. ST contributed to the protocol, designed data collection systems, implemented data management, and helped draft the manuscript. ASV contributed to the protocol and provided advice on operational issues regarding trial design, implemented the trial, and helped draft the manuscript. SVW contributed to the protocol and helped draft the manuscript. CHW conceived and designed the trial, contributed to the protocol, contributed to the design of data collection systems, contributed to the statistical analysis plan, and helped draft and revise the manuscript.

Transparency: AMHR affirms that the manuscript is an honest, accurate, and transparent account of the study being reported; that no important aspects of the study have been omitted; and that any discrepancies from the study as planned (and registered) have been explained.

## Notes

Cite this as: *BMJ* 2015;351:h3740
