# Supplementary material for: The ring vaccination trial: a novel cluster randomised controlled trial design to evaluate vaccine efficacy and effectiveness during outbreaks, with special reference to Ebola
Source: BMJ. 2015 Jul 27;351:h3740. doi: 10.1136/bmj.h3740 (PMC4516343; doi:10.1136/bmj.h3740)
Supplement: Supplementary file 1 [file cama026973.w1_default.pdf]

Protocole d'étude

Essai de vaccination visant à évaluer l'efficacité et l'innocuité du vaccin contre le virus Ebola en Guinée, Afrique de l'Ouest

Partie A : Essai randomisé de vaccination en ceinture visant à évaluer l'efficacité et l'innocuité du vaccin contre le virus Ebola en Guinée, Afrique de l'Ouest

Investigateurs principaux (IP)	Dr Abubakar Soumah<br>Dr Moussa Doumbia<br>Prof Mandy Kader Konde
Promoteur et représentant du Promoteur	Dr Marie-Paule Kieny<br>Sous-Directeur général de l'Organisation Mondiale de la Santé (OMS) pour les systèmes de santé et l'innovation
Fabricants de vaccins	Merck Corporate Headquarters<br>2000 Galloping Hill Road<br>Kenilworth, NJ 07033 U.S.A.

Source de financement	Norwegian Research Council<br>Médecins sans Frontières<br>Wellcome Trust<br>Canadian Public Health Agency<br>Organisation Mondiale de la Sante

Déclaration de confidentialité
Les informations contenues dans ce document sont confidentielles et donc vous sont fournies à titre confidentiel comme examinateur potentiel d'enquêteur.
Il est entendu que ces informations ne seront pas être divulguées à d'autres sans l'autorisation écrite du promoteur, hormis dans la mesure nécessaire pour obtenir le consentement de ces personnes qui peuvent participer dans l'étude.

CONFIDENTIEL

Informations générales de l'étude / structure administrative

**Type d'étude**	Phase 3
**Enregistrement de l'étude**	En cours
**Produit de l'étude**	Vaccin contre le virus Ebola<br>rVSVΔG-EBOV-GP (rVSV)
**Promoteur et représentant du Promoteur**	Dr Marie-Paule Kieny<br>Sous-Directeur général de l'OMS pour les systèmes de santé et l'innovation
**Fabricants de vaccins**	Merck Corporate Headquarters<br>2000 Galloping Hill Road<br>Kenilworth, NJ 07033 U.S.A.
**Source de financement**	Norwegian Research Council<br>Médecins sans Frontières<br>Wellcome Trust<br>Canadian Public Health Agency<br>Organisation Mondiale de la Sante
**Investigateurs principaux (IP)**	Dr Abubakar Soumah<br>Dr Moussa Doumbia<br>Prof Mandy Kader Konde
**Site de l'étude**	A définir, probablement dans la Basse-Guinée
**Moniteurs externe**	Centre pour le développement de vaccins (CVD), Mali<br>University of Maryland<br>AARSH, Senegal
**Comité de surveillance et de suivi**	Pour être mis en place et coordonné par l'Agence de santé publique du Canada
**Vérificateurs externes indépendant**	Rita Walt Consulting

CONFIDENTIEL

Liste préliminaire des institutions et leur rôle dans les essais cliniques proposés.

Institution	Expertise / rôle	Nom
**Investigateurs principaux (IP)**	Epidémiologistes avec l'expérience danse l'essai cliniques et le BPC	Abubakar Soumah<br>Moussa Doumbia<br>Mandy Kader Konde
**Ministère de la Santé publique et l'hygiène (MSPH), et la Division Prévention et la Lutte Contre la Maladie d'Ebola,**<br><br>**République de Guinée**	Les autorités de santé guinéenne feront partie du Comité de pilotage de l'étude. Les autorités de santé guinéenne travailleront en étroite collaboration avec l'INSP, l'OMS et MSF. Les autorités de santé guinéenne contribueront à la mise en œuvre de l'étude, notamment en mettant à disposition des conseils médicaux, des moyens de communication et la propriété des terrains nationaux. En outre, Les autorités de santé guinéenne émettront ses opinions, le cas échéant, sur la mise en œuvre du plan de gestion des données et sur l'analyse statistique.	Investigateurs :<br><br>Mandy Kader Konde (IP)
**Institut national de santé publique (INSP), Norvège**	L'INSP présidera le Comité de pilotage de l'étude, ce qui assurera la coordination du projet. L'INSP travaillera en étroite collaboration avec Les autorités de santé guinéen, l'OMS et MSF.<br><br>En outre, l'INSP émettra son opinion, le cas échéant, sur le développement du protocole d'étude, sur la mise en œuvre du plan de gestion des données et sur l'analyse statistique.	Comité de pilotage de l'étude, Président : John-Arne Røttingen<br><br>Investigateur : Gunnstein Norheim
**MSF Belgique et MSF/ÉPICENTRE**	MSF fera partie du Comité de pilotage de l'étude MSF travaillera en étroite collaboration avec Les autorités de santé guinéenne l'OMS et l'INSP. MSF mènera les opérations d'essai dans le pays, en étroite collaboration avec les autorités guinéennes, l'OMS et d'autres partenaires sur le terrain. En outre, MSF émettra son opinion, le cas échéant, sur la mise en œuvre du plan de gestion des données et sur l'analyse statistique.	Investigateurs :<br><br>Bertrand Draguez<br>Rebecca Grais<br>Abubakar Soumah (IP)

CONFIDENTIEL

Institution	Expertise / rôle	Nom
**Organisation mondiale de la santé (OMS)**	MSF fera partie du Comité de pilotage de l'étude L'OMS travaillera en étroite collaboration avec Les autorités de santé guinéenne, l'INSP et MSF. L'OMS dirigera la gestion des données, l'analyse statistique et la modélisation des activités. En outre, l'OMS coordonnera également les opérations de formation et de monitorage des BPC, le Comité de suivi des données et de la sécurité, et l'audit indépendant de l'essai. L'OMS apportera son assistance pour la mise en œuvre de l'essai sur le terrain, le cas échéant.	Investigateurs :<br>Ana Maria Henao-Restrepo<br>Ximena Riveros<br>Godwin Enwere
**Institut de médecine sociale et préventive (IMSP) et Centres d'essais cliniques (CTU), Université de Berne**	L'IMSP et le CTU (Université de Berne) contribueront au développement du protocole, de méthodes épidémiologiques et d'expertise d'essais ainsi qu'à la gestion de données et à l'analyse statistique des résultats de l'étude.	Investigateurs :<br>Matthias Egger<br>Sven Trelle<br>Stefanie Hossmann
**London School of Hygiene et Tropical Medicine (LSHTM)**	La LSHTM contribuera au développement du protocole, à l'analyse épidémiologique du virus Ebola en Guinée et à l'analyse statistique, et elle coordonnera la modélisation mathématique.	Investigateurs :<br>Conall Watson<br>John Edmunds<br>Anton Camacho
**University of Florida**	L'Université de Floride contribuera à l'élaboration du plan d'analyse statistique, à l'analyse statistique et à la modélisation.	Investigateurs:<br>Ira Longini<br>Natalie Dean
**Centre de développement des vaccins, Mali et Université du Maryland**	Le Centre de développement des vaccins mettra en œuvre la formation au BPC de l'équipe de l'étude et assurera le contrôle de l'essai clinique	Investigateurs :<br>Myron M Levine<br>Moussa Doumbia (IP)<br>Samba Sow<br>Milagritos Tapia
**Public Health England**	La PHE contribuera au développement du protocole, à l'analyse épidémiologique du virus Ebola en Guinée	Investigateur :<br>Sema Mandal
**Agence de santé publique du Canada (ASPC)**	L'ASPC prendra en charge la mise en œuvre sur le terrain et le renforcement des capacités.	Investigateur : À définir
**Merck/NewLink**	Fabricant du vaccin rVSV.	Points de contact :<br>Tom Monath<br>Mark Feinberg

CONFIDENTIEL

Table des matières

1 Page de signature du protocole 8

2 Abréviations 9

3 Contexte 10

3.1 Épidémiologie de la MVE et épidémie actuelle en Guinée 10

3.2 Pratiques exemplaires en matière de prévention, de traitement et de contrôle des MVE14

3.3 Justification de l'étude 14

3.4 Autres études pertinentes en cours 15

4 Plan de l'étude 15

4.1 Conception générale de l'étude 15

4.2 Objectifs de l'étude 18

4.2.1 Objectif primaire 18

4.2.2 Objectifs secondaires 18

4.3 Résultats de l'étude 19

4.3.1 Définition du résultat primaire 19

4.3.2 Définition des résultats secondaires 19

4.4 Taille de l'échantillon 20

4.5 Sélection du site 20

4.6 Surveillance et découverte des cas de MVE 21

5 Organisation de l'étude 22

5.1 Comité de pilotage de l'étude 22

5.2 Coordination de l'étude 24

5.3 Comité de suivi des données et de la sécurité 27

5.4 Chronologie de l'étude 27

6 Population à l'étude et enrôlement des participants 28

6.1 Définition des ceintures de vaccination 28

6.2 Critères d'inclusion et d'exclusion 29

6.2.1 Cas de référence de MVE 29

6.2.2 Population éligible dans les ceintures de vaccination 29

6.3 Obtention du consentement éclairé 30

6.4 Enrôlement 31

7 Vaccination de l'étude 31

7.1 Administration du vaccin 31

7.2 Réduction de la contamination environnementale par les organismes génétiquement modifiés (OGM) 32

7.3 Modifications du dosage 32

7.4 Retrait anticipé de l'étude 32

7.5 Interventions concomitantes 33

8 Informations sur les interventions de l'étude 33

8.1 Interventions 33

8.1.1 Vaccin rVSV contre le virus Ebola 33

CONFIDENTIEL

8.1.2 Résumé des conclusions tirées des études non cliniques 35
8.1.3 Résumé des conclusions tirées des études cliniques 35
8.1.4 Résumé des risques connus et potentiels 35
8.1.5 Stockage et manipulation du vaccin 36
8.2 Procédures d'essai en aveugle 36
8.3 Étiquetage 36
9 Évaluations d'étude 36
9.1 Programme de visite pour ceintures 38
9.1.1 Visite d'éligibilité et de vaccination 38
9.1.2 Visites post-vaccination 39
9.2 Évaluations en cas de retrait anticipé de l'étude 40
9.3 Prestation des soins médicaux 40
9.4 Analyse des MVE en laboratoire 40
9.5 Appel de clôture de l'étude 40
10 Rapport de tolérance pour les événements indésirables graves 40
10.1 Définitions 41
10.2 Évaluation des causes 42
10.3 Rapport d'enregistrement des événements indésirables graves 43
10.3.1 Événements indésirables graves (EIG) 43
10.3.2 Enregistrement des EIG 43
10.3.3 Prise en charge des EIG 44
10.4 Prévisibilité 44
10.5 Système de rapport de l'EIG 44
10.5.1 Rapport initial 44
10.5.2 Rapports de suivi 45
10.6 Grossesse 45
10.7 Déclaration des EIG aux autorités compétentes 46
10.8 Rapports de synthèse 47
11 Statistiques 47
11.1 Randomisation 47
11.2 Analyse statistique 47
11.2.1 Analyse par protocole et par intention de traitement 47
11.2.2 Analyses intermédiaires et règles d'arrêt 48
12 Aspects éthiques et réglementaires 49
12.1 Engagement au sein de la communauté 49
12.2 Approbation du comité d'éthique 49
12.2.1 Déclaration d'Helsinki 49
12.2.2 Directives de l'ICH pour les bonnes pratiques cliniques 49
12.2.3 Approbations 50
12.2.4 Soumission des rapports 50
12.2.5 Dépenses et allocations 50
12.3 Approbation des autorités de réglementation 50
12.4 Enregistrement de l'étude 51

12.5 Consentement éclairé 51
12.5.1 Consentement de la communauté 51
12.5.2 Consentement individuel 51
12.6 Vie privée et confidentialité des participants 52
13 Aspects administratifs 53
13.1 Amendements au protocole 53
13.2 Assurance 53
13.3 Financement et autres soutiens pour l'essai 53
14 Gestion des données et conservation des dossiers 53
14.1 Présentation 53
14.2 Dossier du site de l'investigateur (ISF) 54
14.3 Documents-sources 55
14.4 Tenue des archives 56
14.5 Gestion des données 56
14.5.1 Matériel et logiciel 56
14.5.2 Sécurité, accès et sauvegarde des données 57
14.5.3 Analyses et archivage 57
15 Assurance et contrôle qualité 58
15.1 Validation des données électroniques et centrales 58
15.2 Contrôle sur le site 58
15.2.1 Cadre de monitorage 58
15.2.2 Mise en œuvre des contrôles et des audits sur les sites de l'étude 58
15.2.3 Formation pour assurer la qualité de l'essai 59
15.3 Comité de suivi des données et de la sécurité 59
15.4 Inspections 60
15.5 Clôture ou achèvement de l'étude 60
15.6 Politique de diffusion 60
15.7 Politique de publication et rapport final 60
16 Références 62
17 Annexes 65

CONFIDENTIEL

1 Page de signature du protocole

Révisé et approuvé par la personne suivante :

CONFIDENTIEL

Signature ………………………………………………………….

Dr Marie-Paule Kieny

Sous-Directeur général de l'OMS pour les systèmes de santé et l'innovation

Date…………………16 mars 2015.

Veuillez noter que les responsabilités de l'étude sont documentées dans le dossier local principal de l'essai et de l'investigateur.

2 Abréviations

ASPC	Agence de santé publique du Canada
BI	La brochure de l'Investigateur
BPC	Bonnes pratiques cliniques
BPF	Bonnes pratiques de fabrication
CAd3	Vaccin adénovirus de chimpanzé
CNRS	Comité National d'Éthique pour la Recherche en Santé
CRF	Formulaire d'exposer de cas
CSDS	Comité de suivi des données et de la sécurité
CCS	Comité consultatif scientifique
CPE	Comité de pilotage de l'étude
CTE	Centre de traitement d'Ebola
CT	Centre de transit
CTU	Unité d'essais cliniques
DAT	Équipe de gestion des données et d'analyse statistique
DMST	Équipe de gestion des données et des statistiques
DMC	Centre de gestion des données
DNPL	Direction nationale de la pharmacie et du laboratoire
EI	Evénement indésirable
EIG	Evénement indésirable grave
EC	Comité d'éthique institutionnel
EMA	Agence européenne des médicaments
EOT	L'équipe des opérations de terrain et la mise en œuvre de l'étude
ERC	Comité d'éthique indépendant
FCE	Formulaire de consentement éclairé
FDA	Food and Drug Administration
FLW	Travailleurs de première ligne
FIP	Fiche d'information patient
ICH	Conférence internationale sur l'harmonisation
IP	Investigateur principal
IRB	Comité d'éthique institutionnel
ISF	Dossier du site de l'investigateur
LMIC	Pays à revenu faible et intermédiaire
MSPH	Ministère de la Santé Publique et l'Hygiène
MSF	Médecins sans frontières
MVE	Maladie à virus Ebola
NRA	Autorité nationale de réglementation
OMS	Organisation mondiale de la Santé
ONUSIDA	Programme commun des Nations Unies sur le VIH/SIDA
PCI	Prévention et contrôle de l'infection
POS	Procédures d'opérations standardisées
RPA	Rapport et plan d'analyse
REK	Comité d'éthique régional norvégien
rVSV	Vaccin du virus recombinant de la stomatite vésiculaire
SSL	Secure Sockets Layer
SUSAR	Effet indésirable grave inattendu
TDR	Test diagnostic rapide

CONFIDENTIEL

3 Contexte

3.1 Épidémiologie de la MVE et épidémie actuelle en Guinée

L'épidémie actuelle de la maladie à virus Ebola (MVE) en Afrique de l'Ouest est de loin la plus grosse épidémie jamais enregistrée : un total de 22 092 cas confirmés, probables et suspectés de MVE a été annoncé dans les trois pays les plus touchés (Guinée, Liberia, Sierra Leone, voir figure 3.1) et dans plusieurs autres pays touchés (Mali, Nigeria, Sénégal, Espagne, États-Unis d'Amérique) au 28 janvier 2015, le nombre de décès signalés étant de 8810.[1] L'Organisation Mondiale de la Santé (OMS) a convoqué une réunion d'urgence fin septembre 2014 dans le but d'évaluer les efforts en cours et de tester et produire des vaccins efficaces contre le virus Ebola.[2,3] La réunion a conclu que des essais de phase I devaient être effectués en urgence et que les résultats devaient être largement diffusés afin de faciliter une progression rapide vers les essais de phase II. Des études de phase IIb/III devront être menées en parallèle dans les pays affectés par le virus Ebola, notamment pour protéger les travailleurs de première ligne ou les membres des communautés qui soignent les patients touchés par le virus Ebola dans la communauté ou à domicile.

Les vaccins contre la MVE peuvent réduire le taux de morbidité et de mortalité et mettre un terme à l'épidémie dévastatrice qui affecte le fonctionnement des structures sanitaires. L'étude proposée a le potentiel de contribuer à une meilleure compréhension de l'efficacité et de l'innocuité des vaccins et peut en renforcer l'efficience si une stratégie de vaccination en ceinture est mise en œuvre. L'étude est un effort de recherche multidisciplinaire et collaborative d'urgence face à un défi majeur pour la santé publique et à la crise humanitaire qui s'ensuit.

Maladie à virus Ebola

La maladie à virus Ebola (MVE) est un syndrome fébrile aigu systémique causé par le filovirus Ebola, avec une mortalité entre 30 et 90 % et une période d'incubation entre 2 et 21 jours.[4] Le ratio maladie / infection est généralement décrit comme étant de 1:1, mais certaines infections MVE pourraient également être asymptomatiques.[5] Le taux de mortalité le plus élevé est observé avec le virus Ebola-Zaïre (70 %), le virus responsable de l'épidémie actuelle en Afrique de l'Ouest.[6] La pathogenèse de la MVE est caractérisée par un processus inflammatoire intense, des troubles de l'hémostase et des fuites capillaires, avec une mortalité due à un choc septique et à une défaillance de plusieurs organes.[4] Les signes et symptômes initiaux sont non spécifiques (fièvre, maux de tête, myalgie, fatigue) et peuvent imiter d'autres affections plus courantes, telles que le paludisme. La MVE évolue ensuite vers des symptômes gastro-intestinaux, des saignements internes et externes et, dans certains cas, une éruption cutanée et des troubles neurologiques. Les chauves-souris frugivores, telle *Hypsignatus genus*, sont considérées comme étant le réservoir des filovirus Ebola et de Marburg. L'infection chez l'homme provient du contact avec les excréments ou

CONFIDENTIEL

les fluides corporels des chauve-souris ou d'autres animaux infectés, suivie ensuite d'une transmission homme-à-homme.[7,8] Vingt-cinq épidémies de MVE ont été enregistrées jusqu'à présent. La plus grande épidémie enregistrée avant l'épidémie actuelle était en Ouganda, en 2000 à 2001, avec 425 cas.[9] Dans plus de la moitié des cas, l'origine animale du virus n'a pas pu être prouvée.

Épidémies communautaires

Dans les grandes épidémies, la propagation de l'infection se fait principalement de personne à personne par les contacts physiques directs avec les fluides corporels des patients vivants ou morts atteints d'une MVE. Une étude de la MVE portant sur les membres des familles des foyers affectés, lors de l'épidémie MVE-Kikwit de 1995 en République démocratique du Congo, révélait que sur 27 foyers, 15 (56 %) avaient au moins un cas secondaire et 7 (26 %) avaient plusieurs cas secondaires.[10] Sur les 173 contacts avec des cas primaires au sein du foyer, 28 (16 %) d'entre eux ont développé une MVE. Quatre des 28 cas secondaires n'ont été exposés que pendant la phase de la maladie précédant l'hospitalisation. Les membres adultes de la famille ayant touché le corps du malade ou ayant été en contact avec lui pendant la phase finale de l'hospitalisation étaient exposés à un risque supplémentaire.[10] Il convient également de remarquer que les données de cette étude concernent la période précédant les mesures de précautions strictes instituées dans l'hôpital et dans la communauté.

Groupes à haut risque

Les travailleurs de première ligne (FLW), notamment le personnel soignant (HCW), les membres des équipes d'inhumation et l'équipe d'assistance communautaire, courent un plus grand risque de contracter une MVE. Une analyse des infections menée par l'OMS chez le personnel soignant (rapport non publié) a constaté que les agents de santé représentent 5 % de tous les cas de 15 ans et plus. Le taux d'incidence était plus élevé chez les médecins (39 pour 1000), suivi par les techniciens de laboratoire (29 pour 1000) et les infirmières (25 pour 1000). Globalement, le taux d'incidence parmi le personnel de santé était de 20 pour 1000, par rapport à 0,8 pour 1 000 dans la population générale - soit 25 fois plus de risques. Près de la moitié des infections chez le personnel de santé (42%) se sont produites dans les hôpitaux, les centres de santé et les dispensaires, et seulement 8% dans les CTU, le site de transmission parmi le reste du personnel de santé étant inconnu.

Quatre infections chez des travailleurs de première ligne (FLW) ont été signalées sur la période du 8 au 14 décembre en Guinée. Une étude récente portant sur des patients présentant des symptômes a été menée à Conakry, Guinée, du 25 mars au 26 avril 2014, et a révélé que parmi 37 patients atteints d'une MVE confirmée, 14 (38%) étaient du personnel soignant.[11] Parmi le personnel soignant qui a contracté le virus Ebola, 12 des 14 cas étaient probablement dus à une transmission nosocomiale. Une enquête de cas parmi le personnel de santé à Monrovia, au Liberia, a identifié de multiples possibilités de transmission,

CONFIDENTIEL

notamment l'exposition à des patients avec une EDV non détectée à l'hôpital, l'utilisation inadéquate des équipements de protection individuelle pendant le nettoyage et la désinfection des surfaces ambiantes à l'hôpital, et la transmission potentielle d'un membre du personnel de la santé à un autre.[12]

Immunité protectrice

L'immunité protectrice contre une maladie et infection par le virus Ebola est obtenue par les réponses des anticorps contre les glycoprotéines de surface et par les réponses des cellules T dirigées contre une plus vaste plage d'antigènes viraux. Les anticorps contre les glycoprotéines peuvent empêcher une infection en bloquant la liaison médiateur-récepteur et l'absorption dans les cellules hôtes, tandis que les réponses des cellules T peuvent réduire la sévérité et la mortalité chez les patients infectés. Les lymphocytes T auxiliaires Th1 CD4+ et les lymphocytes T CD8+, qui ont la capacité de tuer les cellules hôtes infectées, peuvent contribuer à la clairance virale.

Les corrélats de la protection basés sur des primates non-humains (PNH) et d'autres modèles animaux incluent les anticorps bloquants et les cellules T capables d'éliminer les cellules hôtes infectées par le virus.[13] Bien qu'un vaccin idéal doive induire une immunité humorale et cellulaire, la production d'anticorps empêchant l'infection est la principale caractéristique d'un vaccin efficace contre la maladie du virus Ebola.

Épidémie actuelle en Guinée

Avec le Liberia et la Sierra Leone, la République de Guinée est l'un des trois pays les plus fortement touchés par l'épidémie du virus Ebola de 2014. On a pu remonter jusqu'au premier cas primaire de l'épidémie qui est apparu à Guéckédou, Guinée, en décembre 2013.[11] Au 28 janvier 2015, la Guinée a rapporté 2917 case d'MVE, dont 1910 décès.[14]

Le taux de mortalité pour les cas avec des résultats connus est de 70 %. À ce jour, la majorité des cas rapportés se situe à Conakry et dans les districts sanitaires voisins des régions côtières et centrales du pays, ainsi qu'en Guinée Forestière, une région montagneuse située au Sud-est de la Guinée, avec les villes de Guéckédou, Macenta et Nzérékoré étant les plus affectées (Figure 3.1).

Au 28 janvier 2015, 162 membres du personnel de santé ont été infectés en Guinée, dont 88 d'entre eux sont morts.[1]

CONFIDENTIEL

Figure 3.1. Districts sanitaires affectés par la MVE (Rapport de situation, OMS, 28 jan. 2015)

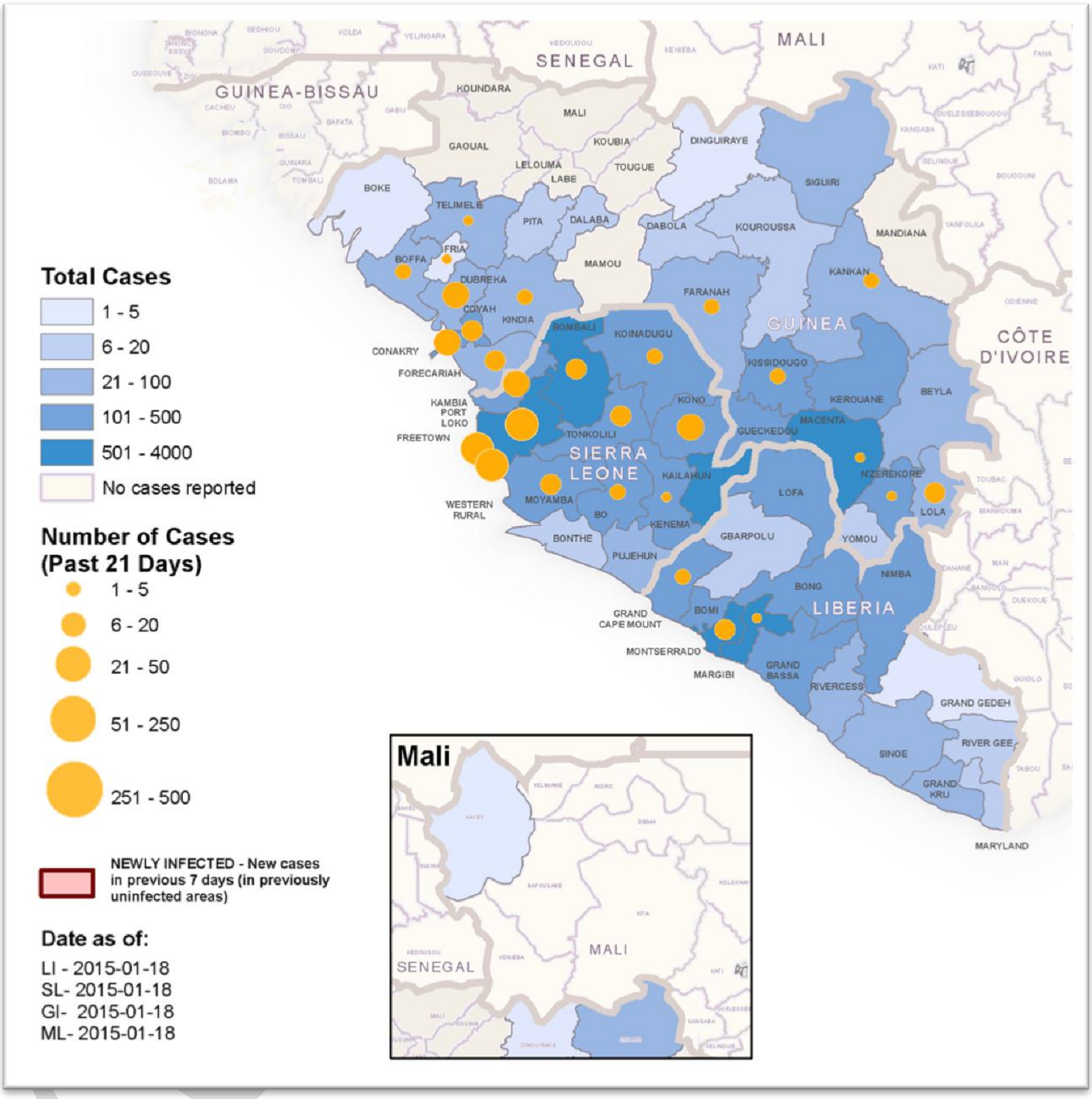

Il existe à présent quatre centres de traitement Ebola (ETC) en Guinée, situés respectivement à Conakry, Macenta et Guéckédou et fournissant un total de 230 lits. Un nouvel ETC a ouvert à Nzérékoré et il est prévu d'ouvrir d'autres ETC à Kérouané, Coyah et Beyla. Dans tout le pays, le nombre de contacts identifiés par cas variait de 2 à 19.[1]

[1] Rapport non publié de l'OMS du 5 décembre 2014

3.2 Pratiques exemplaires en matière de prévention, de traitement et de contrôle des MVE

L'OMS a élaboré des orientations relativement aux meilleures pratiques actuelles adaptées à la Guinée en matière de gestion clinique et de contrôle et prévention des infections.[15–19] En effet, les épidémies précédentes de MVE ont été contrôlées par l'isolement et le traitement efficaces des cas, par une recherche approfondie des contacts, par des pratiques funéraires sûres et par un changement de comportement individuel après une sensibilisation renforcée de la communauté eu égard aux risques.[15] Les directives de l'OMS seront suivies dans la présente étude. En outre, il sera tenu compte des nouveaux éléments concernant les interventions susceptibles d'améliorer la survie, notamment le remplacement des fluides et électrolytes agressifs.[20]

3.3 Justification de l'étude

Il existe un besoin urgent de tester la tolérance et l'efficacité des vaccins proposés actuellement en première phase d'évaluation clinique. Étant donné qu'il n'existe aucun corrélat établi de protection chez l'Homme, des essais d'efficacité sont nécessaires dans le cadre des mécanismes actuels de transmission de la MVE.

La MVE se propage par contact direct avec les fluides corporels des patients symptomatiques. La majorité des contacts sont des membres proches de la famille qui vivent dans le même foyer. Les contacts des personnes venues assister à l'inhumation sont également importants pour la transmission de la MVE, l'exposition ayant lieu à l'occasion de baisers, de la toilette du corps et de contacts physiques avec la personne décédée. La valeur actuelle du taux de reproduction efficace (R_e) de l'épidémie en Guinée est inconnue, mais R_o a été initialement estimé à 1,51.[21] Une vaccination en ceinture consiste en la vaccination ciblée de toutes les personnes vivant dans le voisinage résidentiel et social d'un cas nouvellement diagnostiqué et tout particulièrement des personnes ayant été en contact avec ce cas. Elle crée une zone tampon de personnes immunisées afin de prévenir la propagation de l'infection. Des études de simulation et de terrain montrent que la vaccination en ceinture autour de nouveaux cas peut être efficace et contenir les épidémies de maladies infectieuses avec des taux relativement faibles de reproduction. Des essais contrôlés randomisés dans la population générale, ou des essais sur des groupes géographiquement définis indépendants d'un cas de référence demanderaient des échantillons de grande taille et des durées prolongées d'étude. Inspiré par l'expérience de la variole,[22,23] l'étude de la vaccination en ceinture proposée ici se fera dans une population à risque accru d'infection et fournira des réponses dans un temps relativement court.

Le design choisi est basé sur 3 facteurs : 1) La mise à disposition de preuves claires sur l'efficacité et l'innocuité du vaccin pour l'approbation/ autorisation réglementaire ; 2)La

CONFIDENTIEL

mise en place d'un essai clinique dans une situation épidémique ; 3)La pratique d'un essai clinique contrôlé en Guinée. Un essai clinique à randomisation individuelle, avec un groupe placebo n'est pas accepté. Un design basé sur une comparaison de cas dans le passé n'est pas considéré pertinent dans des conditions épidémiques. Les études par étapes nécessitant un suivi équivalent des nouveaux cas pour toutes les zones ne pourra pas être fait par centre local de surveillance de MSPH/OMS en Guinée. La seule option restante est la vaccination ceintures. Cette vaccination est une forme d'essais cliniques qui a été choisi par sa capacité à fournir des données crédibles dans une situation épidémique. Ce design de vaccination a été utilisé avec succès, en autres, dans le cas de campagnes contre la variole.

3.4 Autres études pertinentes en cours

Deux vaccins proposés sont entrés en étude de phase 1 : le cAd3-EBOV (cAd3) de GlaxoSmithKline (GSK) et de l'U.S. National Institute of Allergy and Infectious Diseases (NIAID), et le rVSVΔG-EBOV-GP (rVSV) de Merck / NewLink Genetics et de l'Agence de santé publique du Canada. Les essais de phase 2 du cAd3 devraient débuter en début 2015, dans quatre pays africains. Il n'existe actuellement aucun autre essai de vaccins concurrents contre le virus Ebola dans la région proposée pour l'étude, nommément la Guinée, en Afrique de l'Ouest. Le premier essai de phase I du vaccin cAd3, une étude à doses progressives menée chez 20 volontaires sains, a été publié le 25 novembre 2014.[24] Les résultats confirmaient la tolérance et l'immunogénicité d'une vaccination unique, avec des réponses des anticorps spécifiques dans la plage rapportée, pouvant être associée à l'immunité protectrice induite par le vaccin lors des tests de provocation chez des primates non-humains.[24] Information additionnelle sur les résultats préliminaires se trouvent dans la Brochure de l'investigateur (BI).

4 Plan de l'étude

4.1 Conception générale de l'étude

L'essai de vaccination en ceinture sera exécuté comme essai de phase 3 randomisé afin d'évaluer l'efficacité, l'effectivité et la sécurité d'une dose de vaccin rVSV dans la prévention des MVE lorsque mis en œuvre comme vaccination en ceinture.

Dans ce modèle d'étude, le cas de référence est un nouveau cas confirmé en laboratoire d'une MVE dans la zone d'étude.

La vaccination en ceinture est une stratégie de contrôle de maladies infectieuses durant laquelle les personnes autour d'un cas sont vaccinées pour les protéger et prévenir une extension de la maladie. Les éléments relatifs à la définition de cette stratégie en ceinture sont détaillés dans les POS 01(v.01) « La définition des ceintures ».

Dans cette étude, la vaccination en ceinture comprends la vaccination de:

CONFIDENTIEL

- Tous les contacts (CF définition ci-dessous a-f) du cas index de MVE, plus
- Les voisins ou les membres de la famille étendue vivant dans la même zone géographique dans laquelle vivent les contacts locaux du cas index, typiquement dans une zone résidentielle délimitée telle une parcelle résidentielle (les délimitations peuvent être un mur, des barrières ou alors la zone est délimitée par un espace libre entre les résidences comme une route, un chemin, des champs ou un foret)
- Des résidents d'une habitation ou vit un contact a haut-risque (CF définition ci-dessous 1-4) qui ne vivent pas dans la même commune que le cas.

La vaccination en ceinture n'est pas nécessairement limitée à un seul site géographique.

Les contacts doivent être identifiés selon les critères suivants:

a) Toutes les personnes ayant vécu avec le cas (vivant ou mort) dans la même habitation depuis l'apparition de la maladie.

(b) Toutes les personnes qui ont visité le malade (vivant ou mort) à son domicile ou dans le centre de santé visité par le patient depuis l'apparition de la maladie.

(c) Toutes les places et toutes les personnes visitées par le malade depuis l'apparition de la maladie (par exemple, le soigneur de médecine traditionnelle, l'église, les proches)

(d) Toutes les centres de santé visités par le patient depuis l'apparition de la maladie, tous les personnels de santé en contact avec le patient (vivant ou mort) sans mesures de protection adéquates

(e) Toutes les personnes en contact avec le corps du patient entre le moment du décès et les cérémonies d'inhumation.

(f) Durant les visite à domicile, les équipes de recherche de contacts et de suivi doivent s'enquérir des personnes qui ont pu être exposées au patient (vivant ou mort) mais qui n'ont pas été identifiées et listées au préalable comme contacts lors des étapes citées ci-dessus.

Parmi ces personnes, les contacts à haut-risque sont définis comme les personnes qui ont lors des 21 derniers jours:

(1) touché les liquides biologiques du patient (sang, vomissures, salive, urine, matières fécales);

(2) été en contact physique direct avec le corps du patient (vivant ou mort);

(3) touché ou nettoyé le linge, les vêtements ou la vaisselle du patient touché ou nettoyé le linge ou les du patient;

(4) Dormi ou mangé dans le même foyer que le patient

Les ceintures de vaccination sont classifiées en urbains ou ruraux.

- Les *ceintures urbaines* sont définies comme ceux de haute densité en population dans les villes en incluant les banlieues.
- Les *ceintures rurales* sont des villages, hameaux et autres endroits non urbanisés.

Si une ceinture est divisée en plus d'un site et inclus à la fois des sites urbains et ruraux, le site doit être classifié selon la localisation de la majorité des cas. Si la répartition des cas est homogène selon les sites, le site contenant l'habitation du cas soit être utilisé pour classifier le site en urbain ou rural.

La détermination d'une nouvelle ceinture de vaccination commence avec la notification par le centre local de surveillance de MSPH/OMS d'un cas confirmé par le laboratoire. L'équipe d'étude doit déterminer si ce cas de MVE est un membre d'une ceinture de vaccination déjà défini auparavant comme décrit dans le POS 02 (v0.1) "Découverte d'un cas (fin de suivi primaire) ''.

L'équipe devra obtenir une copie des informations contenues dans le formulaire d'investigation du cas et les fiches de recherche et suivi des contacts du cas index et utilisera cette information pour initier la saisie de la fiche d'enregistrement de vaccination ceinture.

La ceinture est ensuite randomisé pour recevoir une vaccination immédiate ou retardée et utilisant une POS 15 « La Randomisation ». La vaccination est mise en œuvre d'après les résultats de la randomisation. Au moment de la vaccination, les personnes admissibles dans les ceintures sont listées, puis suivies. L'incidence de la MVE et des évènements indésirables graves (EIG) sera évaluée sur 84 jours par : une surveillance active par des visites planifiées à domicile au jour 0 (vaccination), 3, 14, 21, 42, 63 et au jour 84 ; le rapport des cas de MVE deux fois par jour à partir du centre de surveillance de le MSPH/ OMS pendant les 21 premier jours à partir de l'identification de cas d'MVE ; et la déclaration passive par appel téléphonique par une personne désignée de la population de la ceinture.

Le programme des visites sera identique dans les ceintures vaccination immédiate ou retardée. Le retard dans les ceintures de vaccination retardée sera 21 jours.

La durée de l'étude sur le terrain sera de 8 à 8,5 mois de façon à atteindre les effectifs prévus, incluant la pilotage. L'analyse intermédiaire des résultats d'efficacité sera conduite toutes les 20 ceintures.

CONFIDENTIEL

L'analyse principale mettra l'accent sur l'efficacité du vaccin avec des analyses supplémentaires sur la sécurité et l'effectivité. Les principales analyses pour les objectifs primaires et secondaires seront conformes au protocole. Des analyses en intention de traiter seront également effectuées.

Une orientation appropriée sur un centre de santé pour le traitement du paludisme et d'autres maladies fébriles sera en place, gratuitement au point de livraison.

Figure 4.1. Conception de l'essai de vaccination en ceinture.

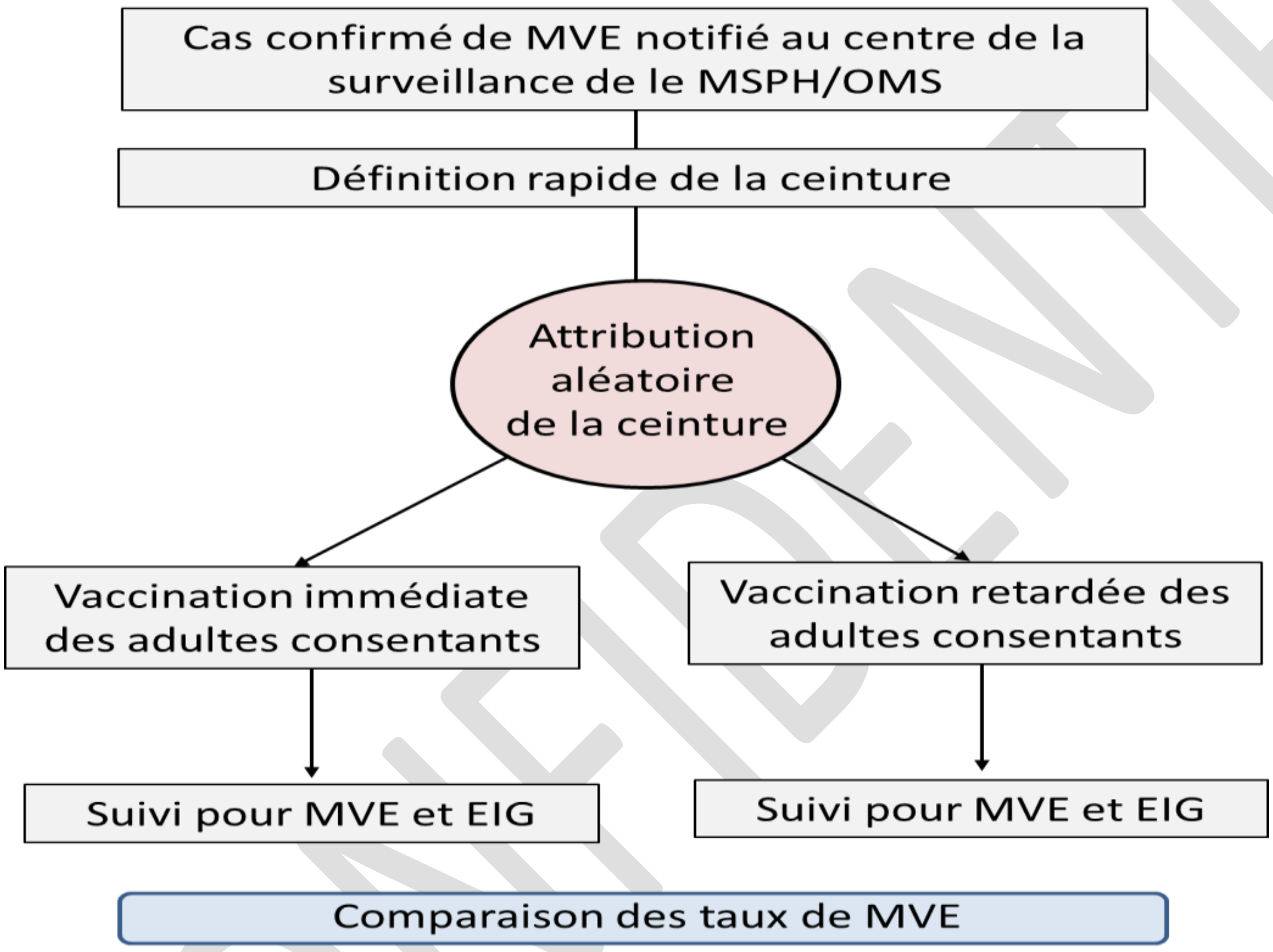

4.2 Objectifs de l'étude

4.2.1 **Objectif primaire**

Évaluer l'efficacité du vaccin contre les maladies à virus Ebola confirmées en laboratoire (MVE) en effectuant un essai clinique comparant la vaccination en ceinture immédiate et retardée.

4.2.2 **Objectifs secondaires**

a) Évaluer l'effectivité globale du vaccin (incidence cumulée) dans la prévention des MVE confirmées en laboratoire au niveau de la ceinture après 84 jours de suivi.
b) Évaluer l'efficacité du vaccin contre la mort par MVE confirmée en laboratoire.

CONFIDENTIEL

c) Évaluer l'efficacité du vaccin contre les MVE probables et suspectées.
d) Évaluer la sécurité du vaccin en suivant les EIG sur 84 jours.

4.3 Résultats de l'étude

4.3.1 **Définition du résultat primaire**

MVE confirmée :

- Tout cas probable ou suspecté (voir définitions ci-dessous) sur lequel un prélèvement de sang a été effectué et est confirmé positif en laboratoire pour la MVE, *ou*
- Toute personne décédée avec une MVE probable et sur laquelle un échantillon post-mortem a été prélevé dans les 48 heures après la mort et confirmé positif en laboratoire pour MVE.

La confirmation en laboratoire est obtenue par essai PCR à transcriptase inverse validée en temps réel (RT) pour l'acide nucléique viral Ebola.

4.3.2 **Définition des résultats secondaires**

MVE probable :

Tout cas suspecté évalué par un médecin OU toute personne décédée suite à une « MVE suspectée » et qui avait une relation épidémiologique avec un cas confirmé, mais qui n'avait pas été testée et n'avait pas eu confirmation de la maladie par des examens de laboratoire.

MVE suspectée :

Toute personne, vivante ou morte, chez qui l'apparition brutale d'une forte fièvre apparaît ou est apparue et qui a été en contact avec un cas suspect, probable ou confirmé de MVE, ou un animal mort ou malade, OU une personne ayant connu l'apparition soudaine d'une fièvre élevée et d'au moins trois des symptômes suivants : maux de tête, vomissements, anorexie / perte d'appétit, diarrhée, léthargie, maux d'estomac, douleurs musculaires ou articulaires, difficulté à avaler, difficultés respiratoires ou hoquet ; OU toute personne ayant des saignements inexpliqués OU toute mort subite et inexpliquée.

Résultats de sécurité :

Les EIG seront évalués. Une description clinique détaillée des EIG devra être fournie à l'aide de formulaires types, concernant notamment les symptômes, la date et l'heure d'apparition, la première observation, le diagnostic, la fin de l'épisode et le résultat final, comme recommandé par la collaboration de Brighton.[25]

4.4 *Taille de l'échantillon*

Sur la base de la littérature et des données inédites sur l'épidémie en cours en Afrique de l'Ouest, nous supposons que 2 % environ des contacts seront infectés et développeront une MVE. Les calculs du nombre de ceintures nécessaires dans chaque bras ont été réalisés pour des taux d'attaque de 1%, 2%, 3%, 4% et 5%, des taux d'efficacité du vaccin de 90%, 70% et 50%, et des tailles de population dans une ceinture de 50 individus, en supposant 20% de perte de suivi, selon l'approche recommandée par Donner et Klar.[26,27] La puissance a été fixée à 90%. Le test statistique utilisé est celui du test bilatéral de Farrington et Manning ; le niveau significatif du test était de 0,05, et le coefficient de corrélation intra-classe de 0,05. Le module d'essai randomisé par groupe avec tests pour deux proportions (en utilisant des rapports) dans le logiciel PASS (version 13, CNSS, Kaysville, Utah, USA) a été utilisé pour les calculs.

Le Tableau 4.1 présente le résultat des calculs. Dans le plus probable scenario, en supposant un taux d'attaque de 2% et un taux véritable d'efficacité du vaccin de 70%, 95 ceintures seront nécessaires dans chaque groupe.

Tableau 4.1. Nombre de ceintures requises dans chaque bras (vaccination immédiate ou différée) en supposant 50 individus par ceinture pour les différentes estimations de l'efficacité du vaccin et une proportion de cas développant une MVE pendant 4 à 6 semaines (voir le texte pour les assomptions).

**Efficacité vaccinale**	**% de cas développant une MVE pendant 1 mois**				
	**1 %**	**2%**	**3%**	**4%**	**5%**
**50%**	*432*	*215*	*142*	*106*	*84*
**70%**	*191*	*95*	*63*	*47*	*38*
**90%**	*98*	*49*	*33*	*24*	*20*

4.5 *Sélection du site*

Le Ministère de la santé et les autres partenaires répondant à l'épidémie prévoient de nouvelles unités de soins et de nouvelles installations d'hébergement pour Ebola, ainsi que l'augmentation du personnel d'action contre l'épidémie dans certaines régions.[28]

En plus d'examiner l'épidémiologie de la flambée des cas, d'autres considérations quant au choix du site incluent la capacité de mettre en œuvre une surveillance adéquate des MVE (comme les guides de l'OMS) dans toute la zone d'étude (comme des installations

appropriées de laboratoire pour les MVE et pour le diagnostic différentiel de la malaria notamment) ; d'autres considérations logistiques (par exemple, état des routes, proximité d'un stockage sûr des vaccins, disponibilité de l'accès aux soins pour la prise en charge des évènements indésirables (EI) et évènements indésirables graves (EIG) potentiels etc.).

Les données épidémiologiques actuelles suggèrent que l'étude sera menée en Basse-Guinée.

Les bonnes pratiques de participation pour la sélection du site seront suivies dans la mesure du possible dans cette situation d'urgence, en prenant en compte les éléments en support de relations constructives avec les acteurs pertinents et les plans appropriées de développement de telles relations, les signes de l'engagement antérieur des acteurs, et la sensibilisation et la prise en compte des problèmes que l'essai pourrait poser en matière de droits de l'homme.[29] Le processus de sélection du site et d'enquêtes d'acceptabilité par la population sera soutenu par les socio-anthropologues et par les experts en matière d'engagement communautaire avec une expérience en Guinée.

Les processus de consentement éclairé prendront en considération les faibles taux d'alphabétisation attendus dans la zone d'étude et les croyances de la population sur l'écriture des noms et des signatures.

Les procédures et outils d'étude éteint mis à l'essai sur plusieurs sites avant la date de début de l'essai, en incluant la capacité de détecter les cas de MVE, la recherche des contacts, la définition de la ceinture, etc.

4.6 Surveillance et découverte des cas de MVE

Les procédures et outils d'étude éteinte mis à l'essai sur sites avant la date de début de l'essai, en incluant la capacité de détecter les cas de MVE, la recherche des contacts, la définition de la ceinture etc.

Tous les cas de MVE identifiés avant la randomisation du cas de référence seront enregistrés et localisés géographiquement et leurs contacts seront identifiés.

De plus, de nouveaux cas de MVE admis dans le Centre de Traitement Ebola (CTE) seront enregistrés dans le centre de surveillance du Ministère de la Santé Publique et d'Hygiène et de l'OMS (MSPH/OMS) et régulièrement confrontés aux listes des personnes éligibles dans les ceintures de vaccination (vaccination immédiate et différée).

Une recherche active des cas de MVE non déclarés sera réalisée par : (i) l'équipe de surveillance MSPH/OMS lors du suivi journalier des contacts de cas pendant 21 jours (ii) l'équipe de l'étude lors des visites de suivi planifiées pour la surveillance des EIG dans les ceintures ; (iii) par des représentants de la population des ceintures. Cette représentation sera faite par une ou plusieurs personnes d'autorité, identifiées pour représenter la ceinture.

CONFIDENTIEL

5 Organisation de l'étude

5.1 Comité de pilotage de l'étude

Cette étude est une collaboration entre partenaires locaux et internationaux (Tableau 5.1). Des Co-investigateurs guinéens supplémentaires seront inclus après l'approbation de l'étude. L'expérience et l'expertise disponibles entre les collaborateurs accéléreront la mise en œuvre de l'étude et faciliteront l'analyse rapide des données et le rapport des résultats.

Tableau 5.1. Liste préliminaire des institutions et leur rôle dans les essais cliniques proposés.

Institution	Expertise / rôle	Nom
Investigateurs principaux (IP)	Epidémiologistes avec expérience dans les essais cliniques et le BPC	Abubakar Soumah<br>Moussa Doumbia<br>Mandy Kader Konde
Ministère de la Santé publique et l'hygiène (MSPH), et la Division Prévention et la Lutte Contre la Maladie d' Ebola,<br>République de Guinée	Les autorités de santé guinéenne feront partie du Comité de pilotage de l'étude. Les autorités de santé guinéenne travailleront en étroite collaboration avec l'INSP, l'OMS et MSF. Les autorités de santé guinéenne contribueront à la mise en œuvre de l'étude, notamment en mettant à disposition des conseils médicaux, des moyens de communication et la propriété des terrains nationaux. En outre, Les autorités de santé guinéenne émettront ses opinions, le cas échéant, sur la mise en œuvre du plan de gestion des données et sur l'analyse statistique.	Investigateurs :<br><br>Mandy Kader Konde (IP)
Institut national de santé publique (INSP), Norvège	L'INSP présidera le Comité de pilotage de l'étude, ce qui assurera la coordination du projet. L'INSP travaillera en étroite collaboration avec Les autorités de santé guinéenne, l'OMS et MSF. En outre, l'INSP émettra son opinion, le cas échéant, sur le développement du protocole d'étude, sur la mise en œuvre du plan de gestion des données et sur l'analyse statistique.	Comité de pilotage de l'étude, Président :<br>John-Arne Røttingen<br><br>Investigateur :<br>Gunnstein Norheim
MSF Belgique et MSF/ÉPICENTRE	MSF fera partie du Comité de pilotage de l'étude MSF travaillera en étroite collaboration avec Les autorités de santé guinéenne l'OMS et l'INSP. MSF mènera les opérations d'essai dans le pays, en étroite collaboration avec les autorités guinéennes, l'OMS et d'autres partenaires sur le terrain. En outre, MSF émettra son opinion, le cas échéant, sur la mise en œuvre du plan de gestion des données et sur l'analyse statistique.	Investigateurs :<br><br>Bertrand Draguez<br>Rebecca Grais<br>Abubakar Soumah (IP)
Organisation mondiale de la santé (OMS)	MSF fera partie du Comité de pilotage de l'étude L'OMS travaillera en étroite collaboration avec	Investigateurs :

CONFIDENTIEL

Institution	Expertise / rôle	Nom
	les autorités de santé guinéenne, l'INSP et MSF. L'OMS dirigera la gestion des données, l'analyse statistique et la modélisation des activités. En outre, l'OMS coordonnera également les opérations de formation et de monitorage des BPC, le Comité de suivi des données et de la sécurité, et l'audit indépendant de l'essai. L'OMS apportera son assistance pour la mise en œuvre de l'essai sur le terrain, le cas échéant.	Ana Maria Henao-Restrepo<br><br>Ximena Riveros<br>Godwin Enwere
Institut de médecine sociale et préventive (IMSP) et Centres d'essais cliniques (CTU), Université de Berne	L'IMSP et le CTU (Université de Berne) contribueront au développement du protocole, de méthodes épidémiologiques et d'expertise d'essais ainsi qu'à la gestion de données et à l'analyse statistique des résultats de l'étude.	Investigateurs :<br>Matthias Egger<br>Sven Trelle<br>Stefanie Hossmann
London School of Hygiene et Tropical Medicine (LSHTM)	La LSHTM contribuera au développement du protocole, à l'analyse épidémiologique du virus Ebola en Guinée et à l'analyse statistique, et elle coordonnera la modélisation mathématique.	Investigateurs :<br>Conall Watson<br>John Edmunds<br>Anton Camacho
University of Florida	L'Université de Floride contribuera à l'élaboration du plan d'analyse statistique, à l'analyse statistique et à la modélisation.	Investigateurs:<br>Ira Longini<br>Natalie Dean
Centre de développement des vaccins, Mali et Université du Maryland	Le Centre de développement des vaccins mettra en œuvre la formation au BPC de l'équipe de l'étude et assurera le contrôle de l'essai clinique	Investigateurs :<br>Myron M Levine<br>Moussa Doumbia (IP)<br>Samba Sow<br>Milagritos Tapia
Public Health England	La PHE contribuera au développement du protocole, à l'analyse épidémiologique du virus Ebola en Guinée	Investigateur :<br>Sema Mandal
Agence de santé publique du Canada (ASPC)	L'ASPC prendra en charge la mise en œuvre sur le terrain et le renforcement des capacités.	Investigateur : À définir
Merck/NewLink	Fabricant du vaccin rVSV.	Points de contact :<br>Tom Monath<br>Mark Feinberg

CONFIDENTIEL

5.2 Coordination de l'étude

Un Comité de pilotage sera soutenu par un Comité consultatif scientifique et par un auditeur indépendant. L'équipe de mise en œuvre des opérations sur le terrain recevra une formation aux BPC et une assistance pour le contrôle de l'étude; l'équipe de gestion des données et des statistiques sera soutenue par le CSDS. Les équipes de terrain seront composées de personnel local et international.

Les comités, leur composition et leurs responsabilités sont établis comme suit :

1) Le Comité consultatif scientifique (CCS) sera composé d'experts dans la conception et la conduite d'essais en Afrique, et dans l'analyse et l'interprétation des essais de vaccination. Le CCS sera composé d'experts qui font actuellement partie du groupe de travail sur le vaccin contre le virus Ebola, mais qui n'auront pas le rôle d'investigateurs dans cette étude.

Un Président sera sélectionné après finalisation de la composition du Comité. Il sera demandé aux membres du CCS de contribuer aux activités suivantes : examen du protocole de l'essai; conseil et accord sur le plan de l'analyse ; réponse rapide aux questions concernant en particulier les méthodes de l'essai, la mise en œuvre pratique, l'analyse et l'interprétation ; examen des projets de rapport final d'essai et des commentaires sur la version finale.

2) Le Comité de pilotage de l'étude (CPE) sera présidé par le Professeur John-Arne Røttingen, de l'INSP. Les autres membres du groupe de pilotage incluront un représentant du Ministère de la Santé de la Guinée et les représentants des promoteurs de l'étude (MSF et l'OMS) et des principaux investigateurs. Afin d'assurer une collaboration étroite avec tous les investigateurs, des réunions et des conférences téléphoniques seront régulièrement organisées. Il sera demandé aux membres de contribuer aux activités suivantes : conseil sur le développement du protocole d'essai, notamment le choix du vaccin, le dosage, les critères d'inclusion et d'exclusion et les indicateurs de résultat ; conseil sur les bonnes pratiques de participation et soutien à l'engagement au côté des acteurs de la collectivité locale, notamment les autorités locales donnant les agréments réglementaires, éthiques et administratifs ; conseil sur les stratégies, priorités, plans de travail et procédures d'opérations normalisées, utilisation des ressources et conseils sur les difficultés survenant pendant la mise en œuvre de l'essai ; amendements et modifications proposés pour traiter ces situations.

3) L'équipe des opérations de terrain et la mise en œuvre de l'étude (EOT) sera responsable de la gestion quotidienne du projet, y compris l'essai de vaccination en ceinture. La EOT développera des plans de mise en œuvre détaillés et organisera, contrôlera et

CONFIDENTIEL

supervisera l'implémentation des activités de terrain de l'étude. L'équipe donnera des conseils sur le suivi et la surveillance des contacts pour la MVE, conformément aux directives de l'OMS[19], et fournira une assistance scientifique et matérielle au laboratoire de l'étude. L'équipe sera responsable de l'implémentation des bonnes pratiques de participation dans les phases préparatoires, pendant la mise en œuvre de l'étude et la diffusion des résultats, sur la base de la directive ONUSIDA développée pour les essais sur le terrain.[29] Elle sera plus particulièrement responsable du développement de la documentation destinée à informer les acteurs et la communauté en général et travaillera aussi en étroite collaboration avec les médias locaux et nationaux, notamment la presse, la radio et la télévision. L'EOT sera organisé en sous-groupes, comme détaillé en Figure 5.1.

4) L'équipe de gestion des données et d'analyse statistique (DAT) développera les plans de gestion et de validation des données ainsi que les protocoles d'analyse statistique et de modélisation d'impact. Elle concevra les CRFs sur papier normalisé et mettra en œuvre le système de gestion de la base de données clinique. L'équipe veillera à ce que les procédures d'opération normalisées appropriées soient révisées et mises à jour. Elle mettra en place, contrôlera et supervisera les activités au centre de gestion des données. Enfin, l'équipe effectuera l'analyse statistique de l'étude et développera un modèle mathématique visant à évaluer l'impact de la vaccination en ceinture contre le virus Ebola, sur la base des données de l'essai.

CONFIDENTIEL

Figure 5.1 L'équipe des opérations de terrain et la mise en œuvre de l'étude (EOT)

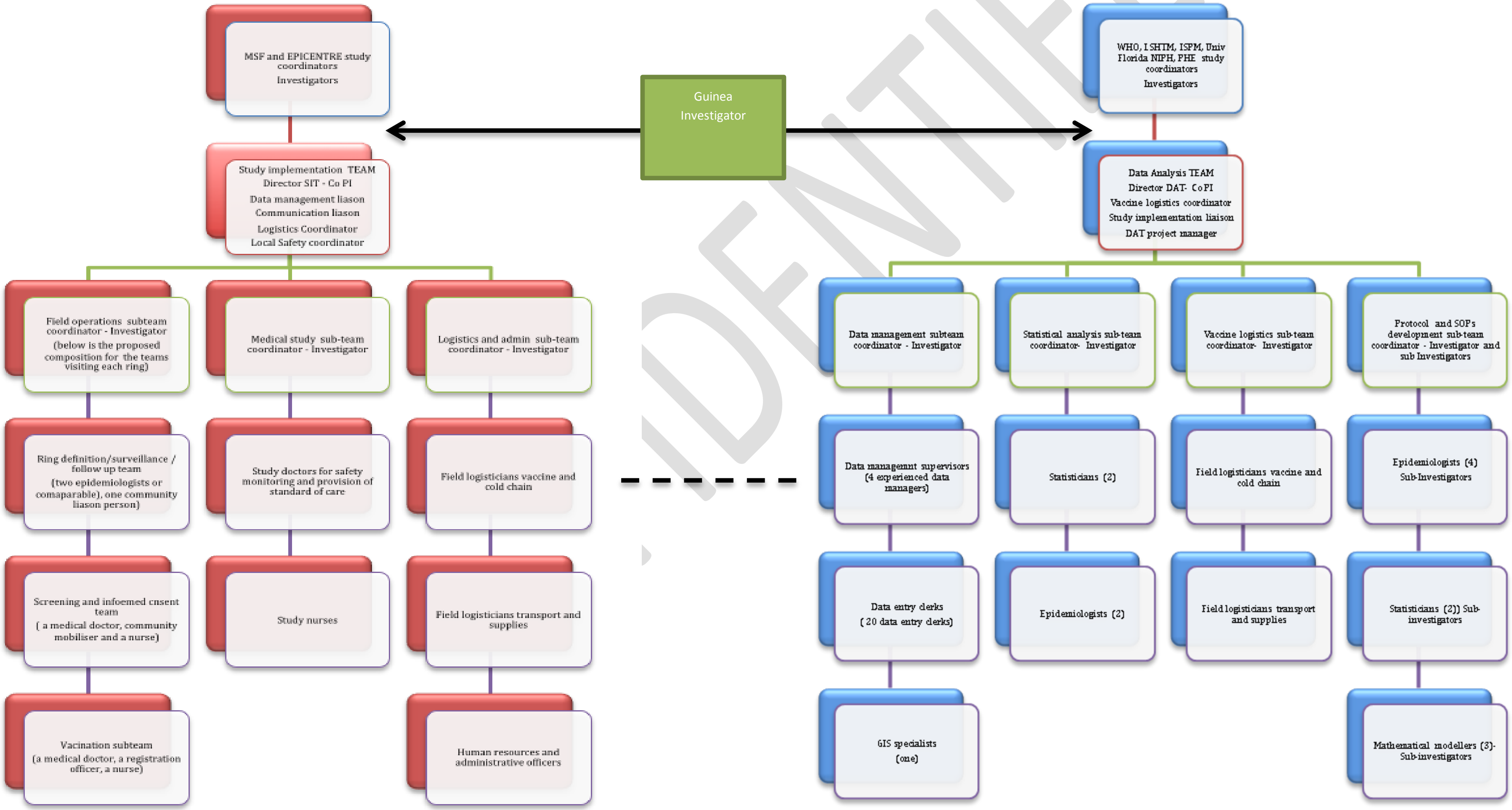

5.3 Comité de suivi des données et de la sécurité

Un Comité de suivi des données et de la sécurité (CSDS) sera instauré avant le démarrage de l'étude. Le comité sera composé d'un groupe d'experts externes à l'essai et indépendants du promoteur chargés de contrôler la conduite de l'essai, d'examiner les données qui en sont tirées, et en particulier les rapports des EIG (associés ou non, indépendamment de la gravité), et d'émettre des recommandations, le cas échéant. Le dossier détaillé ainsi que les directives opérationnelles du CSDS seront définis dans la Charte CSDS. L'étude sera interrompue selon les règles d'arrêt définies à priori. Le CSDS examinera en aveugle les analyses intermédiaires sur base mensuelle.

5.4 Chronologie de l'étude

Le calendrier global pour l'étude s'étalera de décembre 2014 à juin 2017 (31 mois) et les étapes en sont présentées dans le Tableau 5.2.

Tableau 5.2. Étapes des opérations liées à l'étude

**Activité**	**Moment**
Visite initiale au Ministère de la santé de Guinée – examen des données épidémiologiques pour informer de la conception de l'étude	11.2014
Soumission des propositions de financement au NRC, WT	11.2014
Développement des contrats et assurances, charte du CSDS	12. 2014
Désignation du promoteur réglementaire, CSDS, contrôleur	12. 2014
Développement du protocole de l'étude, FCE, IB, IMPD, POS	12.2014
Cartographie rapide des sites et laboratoires potentiels pour diagnostic de la MVE	12.2014
Réunion d'information pour les autorités nationales	12.2014
Visite de suivi au Ministère de la santé de Guinée	01.2015
Engagement de la communauté : Mesures à prendre pour améliorer la participation et la confiance	01.2015
Visite de terrain du MSPH de Guinée – discussion de la faisabilité et de l'appui politique	01.2015
Manuel des opérations pour l'implémentation de l'étude, le plan de formation aux bonnes pratiques cliniques et surveillance	02.2015
Soumissions éthiques et règlementaire aux autorités nationales de Guinée	02.2015
Soumissions éthiques au REK Norvège, OMS et MSF	02.2015
Production du lot de vaccins GMP pour l'essai. Données du CQ sur les lots de vaccins.	01.2015
Libération des lots des vaccins, exportation depuis l'Union européenne / les États-Unis, et produits, expédition, importation en Guinée	02.2015
Choix du vaccin et site de l'étude	02.2015
Évaluation, achat et expédition des articles de l'étude	01.2015
Recrutement et formation du personnel de terrain - construction des capacités du laboratoire et formation aux bonnes pratiques cliniques	02.2015
Visite d'initiation du site	02.2015

CONFIDENTIEL

Activité	Moment
Toutes les préparations complétées pour le démarrage de l'étude :<br>• Agréments éthiques et réglementaires obtenus, communauté sensibilisée, vaccins et kits en place<br>• Infrastructure du site et formation achevées, POS, collecte pilotée des données	02.2015
Premier participant à l'étude recruté et vacciné	28.02.2015
Dernier participant à l'étude recruté et vacciné	06.2015
Analyse intermédiaire	Toutes les 20 ceintures
Analyse des données	10.2015
Rapport final	12.2015

6 Population à l'étude et enrôlement des participants

6.1 Définition des ceintures de vaccination

Après notification par le centre de surveillance du MSPH/OMS d'un cas de MVE confirmé en laboratoire dans le CTE, ou une mort confirmée ou un cas reporté dans la communauté, une liste de contacts sera établie en utilisant les formulaires d'investigation de cas et d'un formulaire de recherche de contacts. L'équipe de recherche travaillera en étroite collaboration avec l'équipe de repérage et de suivi des contacts du centre de surveillance de l'OMS afin d'identifier les contacts au sein de la collectivité, personnes définies selon l'OMS[19] (voir section 4.1)

Dans cet essai, les documents suivants ont été identifiés comme « documents-source »:

1. Fiche d'investigation de cas d'Ebola, OMS
2. Résultats laboratoires Ebola
3. Fiche de recherche des contacts Ebola, OMS
4. Formulaire pour le recensement des contacts des cas de la MVE La liste linéaire), OMS
5. Formulaire de suivi des contacts, OMS
6. Formulaire d'exposé de cas (CRFs)

Le recueil de données avec CRFs est décrit dans les POS 16 (v.01).

Si les informations sont collectées à partir de sources différentes que celles spécifiées plus haut, il sera nécessaire de l'enregistrer et de le documenter.

La randomisation des ceintures entre vaccination immédiate et vaccination retardée sera déclenchée par la notification consécutive de cas confirmes de MVE.

CONFIDENTIEL

Les outils de localisation géo-spatiale seront utilisés pour localiser la situation de la ceinture. Les ceintures devraient comptabiliser aux alentours de 50 personnes mais pourront néanmoins être plus large ou plus petit.

Le processus de définition d’une ceinture sera documenté selon les critères prédéfinis décrits dans les POS Définition des ceintures de vaccination POS 01(v.01). Une fois défini, les personnes appartenant à une ceinture seront visitées à leur domicile pour évaluer leur éligibilité comme décrit dans la section 9.1.

6.2 Critères d'inclusion et d'exclusion

Au niveau de la communauté, les considérations relatives à l'éligibilité incluront l'évaluation des relations significatives avec les parties prenantes concernées et les niveaux d'engagement au sein de la communauté (voir également l'article 12.1). L'éligibilité des nouveaux cas de référence de MVE et des membres des ceintures correspondants sera ensuite évaluée (sur la base des critères ci-dessous), le consentement des intéressés sera demandé et il sera alors procédé à leur enregistrement.

6.2.1 **Cas de référence de MVE**

Tous les cas de MVE nouvellement diagnostiqués et confirmés en laboratoire résidant dans la zone d'étude et n'étant pas résidents dans les ceintures de vaccination définies par d'autres cas de référence seront inclus et traités comme de nouveaux cas de référence. Si les cas de MVE confirmés en laboratoire résident dans une ceinture auparavant randomisé, ils seront comptabilisés comme indicateurs de résultat.

6.2.2 **Population éligible dans les ceintures de vaccination**

Le critère d'inclusion est :

- personnes de 18 ans ou plus vivant dans la ceinture de vaccination définie.

Les critères d'exclusion sont :

- Antécédent de MVE (auto déclaré ou confirmé par le laboratoire).
- Grossesse (signalée oralement) ou allaitement. Les femmes seront invitées, mais pas obligées, à faire un test de grossesse.
- Antécédent d'administration d'autres traitements expérimentaux pendant les 28 derniers jours.
- Antécédent d'anaphylaxie à un vaccin ou un composant de vaccin

CONFIDENTIEL

- Maladie grave clouant la personne au lit ou nécessitant une hospitalisation au moment de la vaccination.

D'autres critères d'exclusion ont été définis selon les résultats des études de phase I et de phase II. Si un participant ne répond pas aux critères d'inclusion, il recevra des conseils de prévention et contrôle des infections (PCI).

6.3 Obtention du consentement éclairé

Au début de l'étude, des opérations de mobilisation sociale et d'engagement de la communauté seront conduites afin d'informer l'ensemble de la population de la zone d'étude sur les objectifs de l'étude.

Une équipe d'opérations de terrain (EOT) visitera la ceinture avec l'équipe de recherche des contacts du MSPH/OMS. Cette EOT est composée par une équipe d'épidémiologistes (définissant la ceinture), l'équipe de consentement éclairé et si la vaccination est immédiate, l'équipe de vaccination.

Si un résident d'une ceinture de vaccination - répond aux critères d'éligibilité, l'équipe d'étude procèdera à la procédure de recherche de consentement avec le participant afin de l'enregistrer en utilisant le formulaire de consentement éclairé**.** La procédure de consentement éclairé et d'enregistrement sera détaillée dans la POS 3 « Consentement éclairé » et la POS 4 « enregistrement du participant », développées en collaboration avec un socio-anthropologue.

La procédure garantira en toute confiance que les éventuels participants comprennent les risques et avantages potentiels, ou le manque d'avantages, du vaccin, les procédures de l'étude (notamment confidentialité et anonymat), leur droit de refuser et/ou de se retirer de l'étude à tout moment sans que cela affecte les services ou les soins de santé qu'ils reçoivent, et sans l'obligation d'exposer le motif de leur refus ou de leur sortie de l'étude.

Il sera demandé aux participants de lire l'intégralité du formulaire de consentement et/ou de recevoir une explication orale complète dans une langue qu'ils comprennent. Dans toutes les ceintures, une réunion sera organisée pour expliquer collectivement le formulaire de consentement éclairé. Il sera demande ensuite à chaque participant individuellement par l'équipe s'ils comprennent toutes les parties du formulaire et la possibilité leur sera donnée de poser des questions et de demander des éclaircissements. Leur consentement sera alors recueilli.

Les participants qui consentent à entrer dans l'étude et à être vaccinés devront suivre le processus de recrutement pour la vaccination et de suivi. Les participants à l'ceinture qui refusent la vaccination seront invités s'ils sont prêts à prendre part à l'étude comme témoin

CONFIDENTIEL

non vacciné. Les personnes qui acceptent d'être recrutées dans le groupe témoin non vacciné seront suivies comme indicateurs de résultat pour la MVE, conformément aux procédures de l'étude.

Si une personne ne souhaite pas être incluse dans l'étude comme participant vacciné ou non (ou comme cas de référence), ils recevront tout de même des conseils en matière de prévention et de contrôle des infections (IPC), ainsi que des soins appropriés en cas de maladie d'eux-mêmes ou de leurs proches. L'équipe de l'étude visera à documenter le nombre de cas de référence et des résidents de l'ceinture avec lesquels contact a été pris, le nombre de personnes refusant de participer, les informations démographiques de base (âge, sexe, mais aucune information d'identification personnelle) et les raisons invoquées pour ces refus. Les personnes peuvent choisir de ne pas divulguer la (les) raison(s) de leur non-participation.

6.4 Enrôlement

Une fois le consentement obtenu, les participants (résidents éligibles dans la ceinture définie) sont inclus dans l'étude. Ils seront informés du calendrier d'évaluation de l'étude, notamment la date de vaccination et les évaluations qui s'ensuivront dans le cadre de l'étude. Une autorité reconnue de la communauté ou un porte-parole établi de la ceinture recevra un téléphone mobile. Les participants seront informés qu'ils peuvent communiquer avec les membres du personnel désignés par l'intermédiaire de cette personne. Ils se verront également donner une carte de contact et ils seront invités à communiquer avec l'équipe de recherche en cas de symptômes préoccupants avant la prochaine évaluation prévue pour l'étude, notamment en cas de fièvre, vomissements, diarrhée, douleurs articulaires etc.

Une copie de la feuille de consentement et une fiche d'information du participant (FIP) seront également remises aux participants avant de conclure la visite d'enregistrement.

La vérification de l'éligibilité, la complétion du consentement éclairé, de la fiche d'enrôlement et la communication de conseils sur le contrôle et la prévention des infections prendront environ une heure et sont détaillées dans la POS 3 « Consentement éclairé « et la POS 4 « Enrôlement du participant ».

7 Vaccination de l'étude

7.1 Administration du vaccin

Le jour de la vaccination, des flacons de vaccin devront être soit décongelés à température ambiante et administrés conformément aux instructions du fabricant et à la logistique locale de l'étude. Le vaccinateur devra porter des gants. Le vaccin sera administré par voie intramusculaire dans le muscle deltoïde de l'un ou l'autre bras. Il est recommandé que

l'injection soit faite dans le bras non dominant. Les procédures pour l'administration du vaccin sont décrites en détail dans la POS 5 « Administration de Vaccin » et les procédures pour la destruction de vaccin sont décrites dans la POS 26.

Pendant l'administration des vaccins, des médecins spécialement désignés pour l'étude et dotés de médicaments essentiels à la survie et d'équipements avancés de réanimation d'urgence devront être disponibles pour la prise en charge des cas de chocs anaphylactiques. Le participant sera observé sur le lieu de vaccination pendant 30 minutes après l'administration du vaccin pour observer d'éventuelles réactions immédiates. Les procédures pour l'observation des réactions immédiates sont décrites en détail dans la POS 6 « Observations post-vaccinale ».

Les procédures d'évaluation de suivi après vaccination sont décrites en détail à l'article 9.

7.2 Réduction de la contamination environnementale par les organismes génétiquement modifiés (OGM)

L'étude sera exécutée conformément aux Règlements britannique de 2014 concernant les organismes génétiquement modifiés (utilisation confinée).[30] Les sites d'inoculation recevront des soins standards post-vaccination, telles que l'application d'une brève pression avec du coton propre ou un pansement adhésif si nécessaire. Le pansement absorbera tous les virus susceptibles de s'échapper à travers l'orifice cutané laissé par l'aiguille et d'être éliminés comme déchets OGM par autoclavage **.**

7.3 Modifications du dosage

Aucune modification de la dose n'est prévue. Une seule dose sera utilisée, laquelle a été établie en fonction du résultat des études de phase 1.

7.4 Retrait anticipé de l'étude

Conformément aux principes de la révision actuelle de la Déclaration d'Helsinki[31] et de toutes autres réglementations, un volontaire a le droit de se retirer de l'étude à tout moment, pour quelque raison que ce soit, et sans obligation de justification de sa décision. **L'investigateur peut exclure le volontaire de l'étude à tout moment, dans l'intérêt de la santé et du bien-être dudit volontaire.** En outre, le volontaire peut se retirer de l'étude pour l'une des raisons suivantes :

- .
- Non-conformité du volontaire aux exigences de l'étude (visites de suivi par exemple).

CONFIDENTIEL

Un EI entraînant l'impossibilité de continuer à observer les procédures propres de l'étude ou la suspension de tout engagement dans l'étude.

Le groupe continuera à suivre toutes les personnes vaccinées, avec leur accord, jusqu'à la fin de l'étude. La raison du retrait sera rapportée dans le CRF, si connue. Si la sortie de l'étude est due à un EI, des visites de suivi ou des soins médicaux appropriés seront organisés, avec l'accord du volontaire, jusqu'à ce que l'EI ait été résolu, stabilisé, ou si une causalité non associée à l'essai a été établie.

7.5 Interventions concomitantes

Toutes interventions requises aux fins du traitement d'une maladie ou d'un état de santé particulier chez un participant enregistré sont autorisée. Les interventions interdites sont les vaccinations avec un vaccin contre le virus Ebola autre que le vaccin expérimental.

8 Informations sur les interventions de l'étude

8.1 Interventions

Deux vaccins candidats sont entrés en études cliniques: le cAd3-EBOV (cAd3) de GlaxoSmithKline (GSK) et de l'U.S. National Institute of Allergy and Infectious Diseases (NIAID), et le rVSVΔG-EBOV-GP (rVSV) de NewLink Genetics / Merck et de l'Agence de santé publique du Canada.[3,32,33].

8.1.1 **Vaccin rVSV contre le virus Ebola**

Vaccin contre le virus Ebola - rVSVΔG-EBOV-GP (rVSV)

Le vaccin Ebola recombiné (rVSV) consiste en un vecteur atténué vivant du Virus de la Stomatite Vésiculaire (VSV) avec suppression de la virulence associée à la protéine VSV G. Cette protéine G est remplacée par la glycoprotéine GP du virus Ebola à partir de la souche Zaïre. La protéine GP est alors présente (et pas seulement exprimée) sur le VSV.

Le virus de la stomatite vésiculaire (VSV) est transmis par les insectes hématophages qui infectent généralement le bétail. L'infection à VSV chez l'homme n'est habituellement pas associée à une maladie grave. L'humain peut demeurer asymptomatique ou présenter une maladie comme la grippe, occasionnellement une rhinopharyngite ou des vomissements, et rarement des vésicules sur la langue ou une mycose vestibulaire. L'encéphalite n'est pas une caractéristique d'infection naturelle de VSV, mais des lésions neurologiques ont été observées dans le bétail et chez les rongeurs et primates non humains (PNH) après

CONFIDENTIEL

inoculation intracérébrale directe de VSV de type sauvage (VSVwt). La neurovirulence du VSVwt dépend strictement de la protéine G VSV : des lésions du tissu nerveux ont été observées dans le PNH dans lequel une injection intrathalamique du virus de type sauvage VSV - mais pas de rVSVΔG-ZEBOV-GP - a été effectué. Ainsi, le risque de neurovirulence après immunisation au ZEBOV-VSVΔG-GP devrait être très faible. De plus, la réplication du virus in vivo VSVΔG-ZEBOV-GP est significativement atténuée par l'échange de la VSV G et de la protéine ZEBOV-G.

Les cellules infectées par la glycoprotéine VSVΔG/ZEBOV produisent de grandes quantités de glycoprotéine d'enveloppe ZEBOV, comme démontré au moyen de la coloration par immunofluorescence, et cela est probablement la base de leur immunogénicité. En effet, les anticorps au ZEBOV-G sont considérés comme essentiels dans la protection contre ZEBOV. La libération d'énormes quantités de glycoprotéine ZEBOV soluble lors d'une infection ZEBOV joue un rôle crucial dans les manifestations de la maladie, notamment avec un dérèglement du processus inflammatoire, la perte de l'intégrité vasculaire et un échappement viral dû à une suppression immunitaire. Les fonctions pathogènes attribuées à la glycoprotéine soluble ZEBOV sont discutées, mais il est probable qu'elle inclut la cytotoxicité, des propriétés immunosuppressives, une perte des propriétés d'adhérence et une dysfonction endothéliale. Pour atténuer ce risque, la glycoprotéine VSVΔG/ZEBOV-GP contient la variante 8A du gène EBOV GP (l'authentique EBOV a 7A en cette position). Cela résulte en une expression exclusive sur toute la longueur de la glycoprotéine et aucune expression de la glycoprotéine soluble.

Dosage

Une dose de $2x10^7$ PFU. La justification du choix de cette dose est la suivante :

1. Alors que des doses plus faibles ont déclenché des réponses immunitaires robustes, et qu’une relation dose-effet a été observée, la dose de $2x10^7$ PFU était statistiquement supérieure à tous les dosages utilises aux études d’épreuve (fin Janvier 2015) un niveau qui est probablement cliniquement significatif à la fois par ***(IgG)*** ELISA et par tests de neutralisation, et ce de manière cohérente selon le type de tests. Cela s’est vérifié au 14 ème et 28 ème jour après vaccination.
2. Il semble ne pas y avoir de relation dose-effet sur la tolérance locale et les réactions systémiques. Bien que des cas d'arthrite et d’arthralgies retardées aient été signalés, ceux-ci étaient généralement légers et transitoires, et cela à tout niveau de dose étudié.
3. Dans des études d’épreuve chez les primates non-humains, un seul singe vacciné est décédé par MVE, dans le groupe de dose $3x10^6$ PFU. Tous les primates non-humains dans des groupes de dose $2x10^7$ PFU et $1x10^8$ ont survécu aux études d’épreuve.
4. La dose de $2x10^7$ PFU peut facilement être produite et peut être fournie en quantité suffisante pour les besoins des études cliniques restantes et pour le lancement de vaccination à large échelle.

Les informations ci-dessous ont été fournies à la FDA / CBER pour révision. L'information sera également soumise à BBIND - 16131 par NewLink Genetics. Pour des renseignements additionnels voir Module 2.

8.1.2 **Résumé des conclusions tirées des études non cliniques**

Des modèles animaux des vaccins du virus Ebola ont été développés chez la souris, le cochon d'Inde, et le hamster syrien.[33] Les vaccins Cad3 et rVSV ont tous deux démontré une protection complète chez ces modèles. Cependant, bien que ces modèles soient importants en tant que contrôle de première ligne pour l'évaluation des vaccins, la valeur prédictive pour la réussite ultérieure du modèle PNH est limitée. Les deux vaccins candidats ont démontré une efficacité de 100 % chez le PNH,[34,35] et il a été démontré chez le PNH que le vaccin rVSV transporte la protection après exposition.[33] Il n’est pas connu si la protection est de longue durée.

8.1.3 **Résumé des conclusions tirées des études cliniques**

Plusieurs essais de phase I chez un petit nombre de volontaires sont en cours ou ont été récemment achevés. Vingt adultes sains ont reçu soit un dosage de 3×10^6 pfu, soit un dosage de 2×10^7 pfu du vaccin rVSV. Aucun problème grave de tolérance n'a été observé, et basé sur les données d'immunogénicité le dosage moins élevée a été choisi pour cet étude. Récemment, un essai de phase I en Suisse sur le vaccin rVSV a été abandonné temporairement à cause de l'apparition d'une légère à importante inflammation des articulations et de manifestations cutanées chez plusieurs participants à l'étude deux semaines après la vaccination.[36] L'essai suisse a repris le recrutement de volontaires depuis le début janvier 2015 avec une dose moins élevée. Il n’est toutefois pas clair actuellement si la réduction de la dose sera suffisante pour éviter les effets observés.

8.1.4 **Résumé des risques connus et potentiels**

La tolérance chez les hôtes immunodeficient, par exemple des patients infectés par le VIH, a été évaluée chez six macaques rhésus infectés par le virus de l'immunodéficience simienne / humaine (VISH). Aucun signe de la maladie n'a été observé chez les six animaux après immunisation par la glycoprotéine VSVΔG / ZEBOV, et quatre des six macaques infectés par le VISH (ceux qui présentaient les numérations de CD4+ les plus élevées) ont été sauvés grâce à l'action de ZEBOV. Le fait que la compétence immunitaire n'est pas requise pour contrôler la glycoprotéine VSVΔG / ZEBOV était confirmé par l'absence de symptômes suite à l'injection de 2×10^5 pfu/ml (soit 10 fois le dosage normal d'immunisation chez la souris) chez des souris gravement immunodeficient. L'absence de neurovirulence de la glycoprotéine VSVΔG / ZEBOV chez les PNH a été démontrée en 2012, comme résumé ci-dessus.

CONFIDENTIEL

8.1.5 **Stockage et manipulation du vaccin**

Le vaccin sera stocké dans un congélateur sécurisé à – 80 °C et transporté à l'aide d'équipements de gestion de la chaîne du froid pouvant maintenir des températures comprises entre -60 °C et -80 °C. Tous les mouvements des vaccins dans le cadre de l'étude seront documentés à l'aide de systèmes de surveillance électroniques. La responsabilité relativement au vaccin, de même que son stockage, son expédition et toute manutention y afférente seront régis par les POS des fabricants. Une chaîne du froid sûre sera établie.

8.2 Procédures d'essai en aveugle

Il s'agit d'une étude ouverte, il sera par conséquent impossible de procéder à l'insu des participants et des investigateurs. L'affectation au groupe immédiate ou à vaccination retardée sera faite en aveugle par le personnel de laboratoire.

8.3 Étiquetage

Étant donné qu'il s'agit d'une étude ouverte, les flacons des vaccins porteront l'étiquette du fabricant et ne seront pas destinés à des participants particuliers.

9 Évaluations d'étude

Aucune évaluation d'étude du cas de référence d'MVE ne sera faite. Les opérations relatives aux visites d'étude activités pour les participants en ceinture sont décrites dans le Tableau 9.1.

CONFIDENTIEL

Tableau 9.1. Activités dans le cadre des visites d'étude au moment de l'enregistrement, le jour de la vaccination et après la vaccination.

Tableau 9.1.a Activités d'étude dans la ceinture de vaccination immédiate

Evaluation	Jour -21	Jour -20 au jour -1	Jour 0 *(vaccination)*	Jour 3	Jour 14	Jour 21	Jour 42	Jour 63	Jour 84
+/- (jours)				+/-1	+/-3	+/-3	+/-5	+/-5	+/-5
Critères d'inclusion et d'exclusion			X						
Consentement éclairé			X						
Visites d'étude			**X**	**X**	**X**	**X**	**X**	**X**	**X**
Recherche et suivi des contacts par l'équipe du MSPH/OMS			Les visites quotidiennes aux contacts						
Surveillance des réactions immédiates post vaccinale			X						
Evaluation des EI non sollicités				X	X				
Évaluation planifiée des EIG			X	X	X	X	X	X	X
Évaluation non-planifiée des EIG						X			

Tableau 9.1.b Activités d'étude dans la ceinture de vaccination retardée

Evaluation	Jour -21	Jour -20 au jour -1	Jour 0 *(vaccination)*	Jour 3	Jour 14	Jour 21	Jour 42	Jour 63	Jour 84
+/- (jours)				+/-1	+/-3	+/-3	+/-5	+/-5	+/-5
Critères d'inclusion et d'exclusion	X								
Consentement éclairé	X								
Visites d'étude	**X**		**X**	**X**	**X**	**X**	**X**	**X**	**X**
Recherche et suivi des contacts par l'équipe du MSPH/OMS	Les visites quotidiennes aux contacts								
Surveillance des réactions immédiates post vaccinale			X						
Evaluation des EI non sollicités				X	X				
Évaluation planifiée des EIG			X	X	X	X	X	X	X
Évaluation non-planifiée des EIG						X			

CONFIDENTIEL

9.1 *Programme de visite pour ceintures*

9.1.1 **Visite d'éligibilité et de vaccination**

Si la ceinture est randomisée sur le groupe à vaccination immédiate (intervention), les membres de la ceinture seront visités chez eux par l'équipe d'étude au même moment que l'équipe de surveillance du MSPH /OMS réalise la première des 21 visites de suivi des contacts du cas. A cette première visite, l'équipe d'étude évaluera l'éligibilité, obtiendra le consentement éclairé et les vaccinera en cas d'éligibilité.

Si la ceinture est randomisée sur le groupe à vaccination retardée, les membres de laceinture seront visités chez eux par l'équipe d'étude au même moment que l'équipe de surveillance du MSPH /OMS réalise la première des 21 visites de suivi des contacts du cas. A cette première visite, l'équipe d'étude évaluera l'éligibilité and obtiendra le consentement éclairé. A la dernière visite (jour 21) de suivi de contact de l'équipe de surveillance du MSPH/OMS, les vaccinateurs de l'équipe d'étude visiteront la ceinture et exécuteront la vaccination de tous participants qui sont éligibles et ont données leur consentement.

Un formulaire normalisé servira à évaluer l'éligibilité selon les critères détaillés dans la section 6.2. Les participantes auront la possibilité, mais non l'obligation, de se soumettre à un test urinaire de grossesse. Selon les directives du ministère guinéen de la santé, les participants fébriles seront examinés au moyen de tests TDR en cas de diagnostic de paludisme et ils seront traités ou orientés comme il convient en fonction de leur état clinique. Aucune autre évaluation de laboratoire de base ne se fera fait. Toutefois, si une MVE est soupçonnée, la personne concernée sera orientée vers le CTE approprié pour subir des tests diagnostiques de laboratoire et pour une prise en charge et un suivi clinique. Le résultat des examens de laboratoires seront suivis.

Après la vaccination, les participants seront observés sur place pendant 30 minutes afin de surveiller tout événement indésirable immédiat suite à la vaccination. Un médecin avec des médicaments essentiels à la survie et des équipements de réanimation d'urgence sera disponible en cas de choc anaphylactique.

Tous les participants recevront des conseils en matière de prévention et contrôle des infections (PCI). En outre, le groupe d'étude assurera la liaison avec le ou les autorités au sein de la communauté dans la ceinture de vaccination afin de renforcer les messages attachés aux conseils en matière de prévention et de contrôle dans la collectivité ; il posera également des questions sur les cas éventuels de MVE au cours des 21 derniers jours.

Les procédures attachées à la visite de vaccination, notamment à l'enregistrement des événements indésirables survenant immédiatement après l'injection, sont décrites dans la POS 6 « Procédure de l'observation post vaccinale » et la POS 7 « Procédure de gestion des réactions immédiates post vaccinales ».

CONFIDENTIEL

9.1.2 Visites post-vaccination

À chaque visite d'étude de suivi, au domicile du participant (0, 3, 14, 21, 42, 63 et 84 jours après la vaccination), le groupe d'étude posera des questions sur les symptômes ou les signes afin de vérifier la présence d'EIG/EI ou de MVE et de tout autre changement dans l'état de santé depuis la dernière visite. La groupe d'étude considéra comme des documents source les informations collectées pendant les visites de suivi quotidienne par l'équipe de surveillance du MSPH /OMS.

En plus de la collecte d'informations sur les EIG, la collecte d'informations non-sollicitées d'EI sera réalisée par l'équipe d'étude le jour 3 et le jour 14. Tous les effets indésirables doivent être suivies jusqu'à la résolution ou la stabilisation et analysé.

Des tests diagnostiques rapides contre le paludisme et des TDR contre la MVE (si disponible et approprié) seront utilisés pour les patients fébriles, puis les patients seront traités ou conviés à des évaluations supplémentaires, comme approprié du point de vue clinique. En cas de suspicion de MVE cliniquement et/ou par le TDR de MVE, le participant sera redirigé vers l'ETC approprié pour des tests de diagnostic en laboratoire et une prise en charge clinique. Le résultat des examens de laboratoires seront suivis.

Il sera rappelé aux participants de contacter le groupe d'étude (par l'intermédiaire du représentant ou du porte-parole désigné pour la vaccination en ceinture ou en appelant le numéro de téléphone inscrit sur leur carte de visite d'étude) s'ils présentent des symptômes d'intérêt entre les évaluations régulières de l'étude. Ils peuvent contacter le représentant de la ceinture à cet effet.

Les participants recevront des conseils en matière de contrôle et de prévention. Le groupe d'étude fera également la liaison avec le chef de la communauté et le personnel de santé dans la ceinture afin de renforcer les conseils en matière de contrôle et de prévention au sein de la communauté et de poser des questions sur les cas possibles de MVE.

Les procédures applicables aux visites post-vaccination, notamment les procédures pour la gestion d'un cas de fièvre ou d'autres EIG ou de MVE éventuelles sont détaillées dans la POS 12 « Visites post vaccination »

Les ceintures à vaccination immédiate et à vaccination retardée feront l'objet de visites le jour -21 (après la randomisation) et 0, 3, 14, 21, 42, 63 et 84 jours après la vaccination. Ces visites reflèteront celles conduites dans les ceintures à vaccination immédiate ou retardée eu égard à la condition des conseils en matière de prévention et de contrôle, à la sensibilisation de la communauté et à la recherche de cas.

9.2 Évaluations en cas de retrait anticipé de l'étude

Les participants qui retirent leur consentement ne recevront pas de visite à domicile mais resteront inclus dans la surveillance des MVE. Il leur sera offert de se soumettre à une évaluation clinique avant de quitter l'étude, conformément aux évaluations faites lors des visites de suivi. En outre, les EIG seront suivis jusqu'à ce qu'ils arrivent à leur conclusion logique, même si le participant est retiré de l'étude. Les participants qui ne font plus l'objet d'aucun suivi seront retrouvés sur la base des coordonnées fournies lors de leur enregistrement.

9.3 Prestation des soins médicaux

Les participants auront accès à des soins médicaux dans le CTE le plus proche pour le diagnostic des MVE. Les procédures applicables au suivi des symptômes de fièvre et de MVE sont détaillées dans la POS 13.

9.4 Analyse des MVE en laboratoire

Les diagnostics pour la vérification des cas de MVE seront effectués dans un laboratoire désigné à Conakry. Les tests seront effectués dans un laboratoire à un niveau de bio tolérance approprié. L'équipe d'étude utilisera les TDR contre la MVE, si disponible et approprié, pour la gestion d'un cas de fièvre dans un participant vacciné. Néanmoins, un cas confirmé est défini comme un résultat positif au test MVE effectué en laboratoire. L'équipe d'étude va obtenir par l'équipe de surveillance du MSPH /OMS, les résultats des examens de laboratoire de cas suspecté, probable ou confirmé de MVE.

9.5 Appel de clôture de l'étude

La visite du jour 84 est la visite de fin de l'étude. L'état de santé général du participant sera enregistré, et les informations sur tous changements de profession ou de résidence du participant, ou sur tout autre facteur important variant au fil du temps, seront collectées lors de cette visite. Les participants seront alors informés qu'aucun autre contact ne sera prévu dans le cadre de l'étude, mais qu'ils devront néanmoins informer par téléphone l'équipe de l'étude de tout événement indésirable grave possible.

10 Rapport de tolérance pour les événements indésirables graves

Les Événements indésirables graves (EIG) seront enregistrés chez tous les participants à l'essai jusqu'à la fin du procès.

CONFIDENTIEL

10.1 Définitions

Un événement indésirable grave (EIG) est toute manifestation indésirable qui :

- entraîne la mort.
- peut menacer la vie : un EI menace la vie si le participant a risqué de décéder pendant sa survenue ; il ne renvoie à aucun événement qui aurait pu éventuellement causer la mort s'il avait été plus grave.
- résulte en une invalidité ou incapacité notable et durable : un EI entraîne une invalidité ou une incapacité si l'événement a pour conséquence une perturbation importante des capacités du participant à exercer ses activités quotidiennes normales. Cette définition n'est pas destinée à inclure des épisodes d'une importance médicale relativement mineure tels que des maux de tête, des nausées, des vomissements, une diarrhée, une grippe, des réactions au site d'injection et un traumatisme accidentel (p. ex. une entorse de la cheville).
- requiert une hospitalisation ou une prolongation d'une hospitalisation en cours : en règle générale, « hospitalisation » signifie que le participant a été retenu (impliquant habituellement un séjour d'au moins 24 heures) à l'hôpital ou aux urgences pour un traitement qui n'aurait pas été approprié dans le cabinet du médecin ou dans un environnement ambulatoire.
- est une anomalie congénitale chez l'enfant d'un participant à l'étude;
- est un événement médical important qui peut compromettre le participant ou qui peut demander une intervention afin d'empêcher que l'un des autres résultats mentionnés ci-dessus soit à considérer comme grave. Des exemples de tels traitements sont le traitement intensif en salle d'urgence ou à la maison pour bronchospasme allergique; dyscrasies sanguines ou convulsions qui ne donnent pas lieu à hospitalisation.

Hospitalisation pour une intervention chirurgicale non urgente associée à un état de santé préexistant dont ni la sévérité, ni la fréquence n'a augmenté après le début de l'étude, ou pour des procédures cliniques de routine (notamment une hospitalisation pour des raisons « sociales ») qui ne sont pas considérées comme des EIG. S'il existe un doute quant à la nécessité de l'hospitalisation qui a eu lieu, l'EI sera considéré comme grave.

La définition d'une procédure clinique de routine est une procédure qui peut avoir lieu pendant l'étude et qui ne doit pas interférer avec l'administration du vaccin, ni avec aucune des procédures spécifiques au protocole en cours. Si quoi que ce soit d'indésirable devait arriver pendant une procédure d'éligibilité et devait satisfaire l'un ou l'autre des critères d'EIG, cette occurrence sera dûment documentée et rapportée.

CONFIDENTIEL

10.2 *Évaluation des causes*

Si un événement répond aux critères à déterminer comme « sérieux » (voir la définition d'EIG), il sera examiné par l'investigateur (et CSDS) afin de déterminer tous les facteurs applicables pour chaque EIG. Ces facteurs contributifs seront documentés et signalés de manière appropriée. L'investigateur devra s'efforcer d'expliquer chaque EIG et d'évaluer sa relation de cause à effet, le cas échéant, avec l'administration du vaccin expérimental.

En cas d'administration concomitante de plusieurs médicaments, il ne sera pas possible de déterminer la relation causale des EIG avec chacun des médicaments administrés. Par conséquent, l'investigateur devra déterminer si l'EIG pourrait ou non être associé à la vaccination plutôt qu'à l'un des médicaments.

Le degré de certitude avec lequel un EIG peut être attribué à l'administration du vaccin à l'étude (ou autres causes alternatives, par exemple l'histoire naturelle des maladies sous-jacentes, le traitement concomitant, etc.) est déterminé selon la façon dont l'événement peut être compris en ce qui concerne une ou plusieurs des opérations suivantes :

- réaction de nature similaire ayant précédemment été observée avec ce type de vaccin et / ou de formulation
- événement ayant souvent été rapporté dans la littérature pour des types de vaccins similaires
- événement ayant été temporairement associé à la vaccination ou reproduit lors d'un rappel de vaccination

L'investigateur évaluera la causalité de tous les EIG, en posant pour cela la question suivante : « Existe-t-il une possibilité raisonnable permettant d'affirmer que l'EIG puisse avoir été causé par le vaccin expérimental ? » Après l'évaluation de la causalité, l'investigateur classera l'EIG comme connexe ou non, tel que défini ci-dessous :

- Connexe : on soupçonne une relation entre le vaccin et l'EIG (sans déterminer le degré de probabilité) ; il existe une possibilité raisonnable que le vaccin ait contribuée à l'EIG.
- Non connexe : on ne soupçonne aucune relation entre le vaccin et l'EIG, il existe d'autres causes plus probables, et rien n'indique que l'administration du vaccin expérimental ait contribué à l'EIG.

CONFIDENTIEL

10.3 *Rapport d'enregistrement des événements indésirables graves*

10.3.1 **Événements indésirables graves (EIG)**

L'enregistrement des événements indésirables graves (EIG) constitue un aspect important de la documentation de l'étude. Il est de la responsabilité de l'investigateur de documenter tous les EIG, conformément aux directives détaillées exposées ci-après. Il sera demandé à tous les participants de contacter immédiatement l'investigateur en cas d'apparition de signes ou de symptômes perçus comme graves pendant la période comprise entre le recrutement et la fin de l'étude. Un contrôle devra être effectué pour les événements qui exigent un traitement ou une hospitalisation, ou qui ont entraîné un décès. Chaque fois que possible, les EIG devront être documentés en termes de diagnostic ou de syndrome, plutôt que par plusieurs symptômes qui sont clairement des manifestations du même diagnostic / syndrome. Si le participant rapporte des signes et symptômes, l'investigateur devra établir un diagnostic médical. S'il est impossible d'établir un diagnostic, entrez alors chaque signe ou symptôme comme des événements séparés. Les conditions, signes et / ou symptômes préexistants (notamment ceux qui n'ont pas été reconnus à l'entrée dans l'étude mais qui l'ont été ultérieurement pendant la période de l'étude) présents chez un participant avant le recrutement devront être enregistrés dans le formulaire des antécédents médicaux, que vous trouverez dans le CRF du participant. Les effets indésirables graves qui surviennent après que le participant ait été enregistré dans l'étude, mais avant la première vaccination, seront versés aux antécédents médicaux et aux sections relatives aux conditions préexistantes du CRF et peuvent requérir un report ou une annulation de la vaccination pour ce participant.

10.3.2 **Enregistrement des EIG**

Les EIG de tous les participants seront collectés au moyen d'un formulaire EIG (questionnaire structuré) renseigné lors des évaluations planifiées de l'étude et dans les rapports établis par le participant entres deux visites d'évaluation. Les EIG seront suivis jusqu'à leur résolution, l'arrêt du traitement ou la fin de l'étude, indépendamment de la gravité et du fait qu'ils soient considérés comme associés ou non à la vaccination.

Les EIG observés par les investigateurs ou rapportés par le participant ou sa famille devront être évalués par l'investigateur et enregistrés dans la page EIG de la CRF du participant. Le diagnostic, la date et l'heure (si approprié) de l'apparition, la conséquence, la gravité et la relation avec la vaccination devront être établis. Les détails de tout traitement administré seront enregistrés de la façon qui convient.

Le rapport initial d'EIG sera suivi d'un résumé complet rédigé en utilisant le formulaire de Rapport d'EIG détaillant les aspects pertinents des événements indésirables en question. Si applicable, les informations contenues dans les dossiers hospitaliers et les rapports

CONFIDENTIEL

d'autopsie des patients concernés devront être obtenues. Les informations supplémentaires ou mises à jour de suivi devront être transmises au coordinateur de pharmacovigilance dès que possible, et au plus tard cinq jours calendaires après la réception des nouvelles informations.

10.3.3 **Prise en charge des EIG**

L'EIG sera pris en charge conformément aux bonnes pratiques médicales actuelles. La gestion de tout EIG sera enregistrée dans le formulaire d'expose de cas. Les services d'assistance médicale, de consultation et de traitement médicamenteux seront fournis à l'ETU du site d'étude. Les participants seront également orientés vers l'établissement ou le système de santé local et traités conformément aux règlements sanitaires nationaux.

10.4 Prévisibilité

Les EIG seront considérés comme inattendus si la nature, la gravité ou la conséquence de l'événement n'est pas en accord avec les informations actuellement disponibles sur le vaccin, comme documenté dans les Informations de référence relatives à la tolérance.

10.5 Système de rapport de l'EIG

10.5.1 **Rapport initial**

Tout événement indésirable qui se qualifie comme événement indésirable grave, qu'il soit ou non considéré comme associé au vaccin expérimental, devra être rapporté au coordinateur de pharmacovigilance par téléphone ou par courriel dans un délai de 24 heures (1 jour calendaire) après que le site ait été informé de l'événement. Si l'EIG est mortel ou met la vie en danger, ou est un effet indésirable grave inattendu (SUSAR), le coordinateur de pharmacovigilance devra être informé immédiatement par téléphone, en plus du formulaire dûment rempli.

L'investigateur principal (ou son délégué) devra documenter toutes les informations disponibles à propos de l'EIG sur le formulaire Rapport d' EIG, puis envoyer ledit rapport par télécopie / courriel au coordinateur de pharmacovigilance, avec copie au fabricant du vaccin. L'envoi de la télécopie ou du courriel pourra être suivi d'un appel téléphonique confirmant la réception du formulaire de Rapport d'EIG. Lorsqu'il remplira le rapport d'EIG, l'investigateur principal (ou son délégué) devra veiller à donner autant d'informations que possible. Le champ de l'investigateur pour l'évaluation de la causalité ou de la parenté avec le vaccin expérimental devra être rempli (non laissé en blanc) après discussion préalable (avec l'IP en cas de délégation) si possible dans le rapport initial. L'investigateur principal (ou son délégué) devra veiller à ce que les informations reportées sur le formulaire soient lisibles, ne comportent pas d'abréviations ni d'identifiants personnels et institutionnels, et soient aussi complètes que possible.

CONFIDENTIEL

Les éléments suivants sont les informations minimales requises pour le rapport initial d'EIG :

- numéro du participant, date de naissance
- description de l'événement
- date de la vaccination
- informations sur le déclarant
- évaluation préliminaire de la causalité
- gravité

Un rapport plus détaillé de l'événement indésirable grave devra suivre le rapport initial d'EIG envoyé au coordinateur de pharmacovigilance, dans un délai de cinq jours après la réception des nouvelles informations. L'investigateur principal (ou son délégué) devra vérifier que tous les événements indésirables graves sont reportés dans la documentation source (c.-à-d. le dossier médical du participant) et dans le CRF. L'investigateur principal (ou son délégué) devra également conserver un enregistrement de toutes les communications concernant l'EIG envoyées au coordinateur de pharmacovigilance ou au fabricant du vaccin.

10.5.2 Rapports de suivi

Les EIG seront suivis jusqu'à ce qu'ils en arrivent à leur conclusion, même si le participant est retiré de l'étude, ou l'étude de suivi soit complétée. Les participants qui ne font plus l'objet d'aucun suivi sont activement recherchés. Les conclusions possibles d'un EIG incluront : le suivi jusqu'à sa résolution (la santé du participant est revenu à son état de base) ; le suivi jusqu'à sa stabilisation (aucune autre amélioration ou aggravation de l'événement n'est prévue); ou toute autre explication de l'événement. Tous les effets indésirables doivent être suivies jusqu'à la résolution ou la stabilisation et analysé et seront analysé à la fin de l'étude.

Un rapport de suivi contiendra les nouvelles informations ou des informations mises à jour ou corrigées. Le rapport de suivi établira si l'événement a été résolu ou se poursuit, s'il a été traitée et la manière dont il l'a été, et il inclura la documentation de toutes les mesures de soutien prises.

Tous les EIG dont l'investigateur a connaissance et qui surviennent à tout moment hors de la période de l'étude et sont considérés comme causés par le vaccin expérimental – et par conséquent codifiés comme de possibles EIG causés par le vaccin - devront être rapportés au coordinateur de pharmacovigilance et au fabricant du vaccin.

10.6 Grossesse

Afin d'assurer la sécurité de la participante et de l'enfant à naître, chaque grossesse déclarée par une participante qui a été vaccinée pendant l'essai devra être rapportée au promoteur dans un délai d'une semaine après en avoir eu connaissance au moyen du Formulaire de déclaration de grossesse.

CONFIDENTIEL

La grossesse sera suivie afin d'en établir le résultat, notamment l'arrêt spontané ou volontaire de grossesse, les détails de naissance, la présence ou l'absence de malformations congénitales, ou les éventuelles complications pour la mère et / ou le nouveau-né. Le suivi de grossesse se fera même si la durée de sécurité prévue pour le suivi dans le cadre de l'essai a pris fin.

10.7 Déclaration des EIG aux autorités compétentes

Le coordinateur de pharmacovigilance sera chargé de la coordination du contrôle et du rapport de tolérance pendant l'étude.

L'investigateur principal signalera les EIG à l'IRB / EC selon le droit local et selon les exigences de l'IRB / EC. L'investigateur principal les signalera également au fabricant du vaccin. Une documentation adéquate sera fournie au promoteur, montrant que l'IRB / EC et le fabricant ont été correctement informés.

Le coordonnateur de Pharmacovigilance signalera les EIG à l'ERC de l'OMS et au CSDS, comme requis. Une description détaillée des fonctions et responsabilités du CSDS, ainsi que les directives sur le flux de transmission des EIG, est donnée dans la charte CSDS. La notification des EIG aux autorités nationales de réglementation compétentes devront suivre les exigences légales nationales.

Le Tableau 10.1 résume le calendrier des rapports d'EIG.

CONFIDENTIEL

Tableau 10.1. Calendrier des rapports d'EIG

Rapporté à	Rapporté par	Délai
Coordinateur de pharmacovigilance	Investigateur principal (ou son délégué)	Dans les 24 heures pour tous les EIG
IRB local	Investigateur principal (ou son délégué)	Dans les 24 heures pour tous les EIG
CSDS	Coordinateur de pharmacovigilance	Dans les 24 heures pour les événements mortels ou mettant la vie en danger ou SUSAR, et dans les 7 jours pour les autres SUSAR (conformément à la charte CSDS)
Fabricant du vaccin	Investigateur principal (ou son délégué)	Dans les 24 heures pour tous les EIG
ERC OMS, CSDS, investigateurs, fabricants	Coordinateur de pharmacovigilance	Synthèses mensuelles des EIG

10.8 Rapports de synthèse

Depuis le début de l'étude jusqu'à la visite de fin du dernier participant de l'étude, le coordinateur de pharmacovigilance devra fournir un rapport de synthèse mensuel actualisé. Ce rapport devra résumer les données des EIG en mentionnant le nombre de cas, les EIG déclarés (diagnostic), le délai d'apparition, la causalité et le résultat. Cette synthèse devra être envoyée aux membres du CSDS, aux investigateurs, à l'ERC / IRB et au fabricant du vaccin. Le CSDS peut également demander un rapport spécial de synthèse des EIG mis à jour.

11 Statistiques

11.1 Randomisation

La randomisation sera réalisée au niveau des ceintures (cf Définition dans les POS 15) et non pas au niveau individuel des participants.

La liste de randomisation sera générée par un statisticien indépendant qui ne sera pas impliqué par ailleurs dans l'étude.

La liste sera conservée en sécurité dans un système d'information électronique avec accès restreint (les personnes impliquées dans le recrutement n'auront pas de droit d'accès).

L'allocation des sujets ne sera visible pour une ceinture donnée uniquement lorsque la ceinture sera enregistrée.

Pour permettre la confidentialité de la randomisation jusqu'à ce que tous les consentements éclairés auront été obtenus pour une ceinture, des enveloppes inviolables, opaques et scellées seront utilisées, ainsi que des containers scellés pouvant contenir les flacons de vaccins (ou pas selon les résultats de la randomisation, cf POS pour plus de détails).

11.2 Analyse statistique

11.2.1 Analyse par protocole et par intention de traitement

Le résultat principal de l'efficacité du vaccin contre la MVE (VE) sera estime en fonction de $\widehat{VE_S} = 1 - \hat{\theta}$ où $\hat{\theta} = \widehat{\lambda_1}/\widehat{\lambda_0}$, le ratio de risque pour la MVE

$\widehat{\lambda_1}$ = le risque estimé d'une MVE pour les personnes dans la ceinture recevant une vaccination immédiate.

$\widehat{\lambda_0}$ = le risque estimé d'une MVE pour les personnes recevant une vaccination différée, avant que les personnes dans la ceinture concernée reçoivent le vaccin.

CONFIDENTIEL

Ce ratio de risque sera estimé en utilisant pour cela un modèle régressif Cox à effets mixtes avec effet aléatoire (fragilité), temporellement dépendant pour la ceinture de vaccination. La vérification d'hypothèse pour le résultat principal sera H_0 : $VE_S = 0$ par rapport à H_a : $VE_S \neq 0$. De plus, un intervalle de confiance de 95% sera calculé pour $\widehat{VE_S}$.

Pour décrire ce modèle d'analyse, nous suivons les méthodes de données de survie groupées présentées dans le chapitre 7 dans Aalen, et al (2008).[38] Des ceintures M seront définies, et randomisées sur l'un des deux groupes de vaccination (v = 0 : retardée ; v = 1 : immédiate). Soit N_{hv} la taille du groupe de vaccination v en ceinture h. La fonction de risque pour la personne i^{th} avec un groupe de vaccination v en ceinture h est

$$\lambda_{hvi}(t) = Z_h \lambda_0(t)\, Y_{hvi}(t) \theta^v e^{X_{hvi}(t)'\beta}, i = 1, \cdots, N_{hv}, v = 0{,}1, h = 1, \cdots, M \qquad (1)$$

Z_h représente la variable aléatoire de regroupement avec une moyenne 1 et une variance σ, et $X_{hvi}(t)$ représente un vecteur de Co variables (par ex., âge, sexe, niveau d'exposition) et β le vecteur de coefficients associés aux Co variables à estimer. De plus, nous définissons l'indicateur d'exposition au risque en fonction du temps $Y_{hvi}(t)$ pour la personne i en ceinture h au moment t. Ce modèle suppose que l'efficacité du vaccin est le même dans tous les ceintures vaccinés.

Les données requises pour conformation à ce modèle sont les moments d'apparition des MVE ou les temps de censure à droit, c'est-à-dire, aucun cas, dans l'ceinture h, défini par $H_h = \{\tilde{T}_{hvi}, D_{hvi}; i = 1, \dots\dots, N_{hv}, v = 0{,}1\}$, avec D_{hvi}= 1 si la personne i est un cas et D_{hvi}= 0 si elle ne l'est pas, et $\tilde{T}_{hvi}$ est le moment de l'apparition du cas si D_{hvi}= 1, ou le temps de censure si D_{hvi}= 0. Selon la variable aléatoire de regroupement Z_h, la contribution de probabilité pour l'ceinture h^{th} dans le modèle à effets mixtes est

$$Pr(H_h|Z_h) = \prod_{i=1}^{N_{h0}} \left\{ \left(\lambda_{h0i}(\tilde{T}_{h0i})\right)^{D_{h0i}} exp\left(-\Lambda_{h0i}(\tilde{T}_{h0i})\right) \right\}^{Y_{h0i}(0)} \times \prod_{i=1}^{N_{h1}} \left\{ \left(\lambda_{h1i}(\tilde{T}_{h1i})\right)^{D_{h1i}} exp\left(-\Lambda_{h1i}(\tilde{T}_{h1i})\right) \right\}^{Y_{h1i}(0)} \qquad (2)$$

avec $\Lambda_{hvi}(T) = \int_0^T \lambda_{hvi}(t)dt$ représentant la fonction de risque cumulatif de (1). En supposant que Z_h suit une distribution gamma, les paramètres $VE_S = 1 - \theta$, β et σ seront estimés et des tests de vérification seront effectués en utilisant le logiciel de survie détaillé dans Munda, et al. (2012).[39] Les analyses intermédiaires et les règles d'arrêt seront appliquées selon les méthodes standards.

Tous les détails seront fournis dans le Plan d'Analyse Statistique.

11.2.2 Analyses intermédiaires et règles d'arrêt

Cet essai sera conduit selon un mode adaptatif avec une cible probable aux alentours de 100 ceintures randomises de vaccination précoce ou tardive. Le nombre total de nouveaux cas est estime aux alentours de 50 (CF section sur la taille de l'échantillon ci-dessous pour plus

CONFIDENTIEL

de détails). Nous utiliserons un test avec hypothèses séquentielle et la réduction de **α** (par la multiplication des tests statistiques). Il est prévu de conduire des analyses intermédiaires des données tous les 20 ceintures randomisées, avec 2-3 nouveaux cas supplémentaires à chaque analyse. A chaque analyse, la décision de continuer ou pas l'essai sera prise par le Comité de Suivi des Données et de la Sécurité (CSDS) selon l'occurrence de prévue de succès, d'échec ou d'insuffisance de preuves. L'essai continuera jusqu'à ce qu'il soit arrêté comme décrit ci-dessus ou s'il 'y a suffisamment de ceinture nouvelle de recrutement.

Les directives relatives à l'interruption de l'étude seront incluses dans la Charte de CSDS et dans le Plan d'analyse statistique.

12 Aspects éthiques et réglementaires

12.1 Engagement au sein de la communauté

Un engagement approprié au sein des communautés de la zone couverte par l'étude sera crucial. Nous appliquerons les directives élaborées par l'ONUSIDA pour la recherche par essai clinique afin d'assurer la participation et le respect mutuel des valeurs et des actions.[29] Nous demanderons conseil aux représentants de la communauté avant, pendant et après l'essai, garantissant ainsi un processus participatif transparent et constructif.

L'étude sera discutée avec les dirigeants de la communauté, et il leur sera demandé d'exprimer leurs avis sur la manière de gérer au mieux la mise en œuvre. Des explications claires et appropriées seront données à la communauté.

Les dirigeants de la communauté seront informés des avantages de l'étude et du fait que les données recueillies seront précieuses pour le développement du vaccin contre le virus Ebola et pourraient contribuer au contrôle de l'épidémie Ebola, avec la possibilité de réduire le taux de morbidité et de mortalité.

12.2 Approbation du comité d'éthique

12.2.1 **Déclaration d'Helsinki**

L'investigateur devra veiller à ce que l'essai soit mené conformément aux principes de la Déclaration d'Helsinki.

12.2.2 **Directives de l'ICH pour les bonnes pratiques cliniques**

L'investigateur devra veiller à ce que l'essai soit mené en totale conformité avec les réglementations applicables et avec les directives de l'ICH relatives aux bonnes pratiques cliniques (CPMP/ICH/135/95) de juillet 1996.[40]

12.2.3 **Approbations**

Les problèmes éthiques associés à la conception de l'étude pour les essais sur les traitements de la MVE ont été discutés par le Groupe de travail de l'éthique de l'OMS lors d'une réunion qui s'est tenue à Genève du 20 au 21 octobre 2014 et ont été pris en compte pour la conception de l'étude proposée.[41]

Le protocole, le formulaire de consentement éclairé, la fiche d'information patient et tout matériel publicitaire proposé seront soumis pour examen éthique aux comités éthiques suivants :

- Comité national d'éthique pour la recherche en santé (CNRS), Conakry, Guinée
- Comité d'éthique indépendant de l'OMS (ERC OMS), Genève, Suisse
- Médecins sans frontières (MSF), Comité d'éthique
- Comité d'éthique régional norvégien (REK), Oslo, Norvège

En raison du besoin de mise en œuvre rapide de l'essai clinique, les candidatures seront soumises simultanément à ces comités. L'investigateur devra soumettre et, le cas échéant, obtenir l'approbation des parties citées ci-dessus pour tout amendement important aux documents originaux approuvés.

12.2.4 **Soumission des rapports**

Les investigateurs devront, une fois par an pendant l'essai clinique, ou sur demande, soumettre le rapport annuel de progression aux comités d'éthique appropriés, à l'organisation hôte et au promoteur. En outre, une notification de fin de l'essai et un rapport final devront être soumis aux mêmes comités et organisations.

12.2.5 **Dépenses et allocations**

Il n'est pas prévu que les participants se déplacent pour des visites d'étude, celles-ci devant être faites à domicile. Cependant, si cela se produisait, des frais de déplacement raisonnables pour toutes visites en plus des soins normaux seront remboursés sur présentation des reçus, ou une allocation au kilométrage sera accordée, comme approprié.

12.3 Approbation des autorités de réglementation

L'approbation réglementaire pour l'essai du vaccin sera obtenue de la Direction Nationale de la Pharmacie et du Laboratoire (DNPL). L'approbation des autorités de réglementation impliquera les directives et normes suivantes :

- Les exigences définies par l'Autorité nationale de réglementation (NRA) et par le Ministère de la santé publique et l'hygiène (MSPH) de Guinée pour approbation de l'essai clinique.

CONFIDENTIEL

- Le format de la documentation pour la demande d'essai clinique respectera la version la plus récente des directives des BPC de l'ICH.

L'approbation réglementaire par la NRA guinéenne sera soutenue par Santé Canada après la demande des autorités de Guinée.

Le niveau de documentation de l'étude permettra donc l'utilisation des données de cet essai dans le cadre de l'homologation potentielle des vaccins, soit par la FDA, soit par l'EMA.

12.4 Enregistrement de l'étude

Avant le début de l'étude, celle-ci sera enregistrée dans le registre panafricain d'essai clinique (selected.pactr.org) qui est en outre indexé par la plateforme du registre international des essais cliniques (selected.who.int/ictrp/en/).

12.5 Consentement éclairé

12.5.1 **Consentement de la communauté**

Le processus de participation communautaire fera un état de la volonté et des exigences de la communauté en termes de participation à l'étude, ceci afin d'assurer la participation et le respect mutuel des valeurs et des actions. Une équipe d'étude rendra visite aux communautés sélectionnées pour discuter du consentement de la collectivité avec les parties prenantes.

12.5.2 **Consentement individuel**

Les participants éventuels se verront expliquer l'étude en profondeur et, en outre, ils se verront remis une fiche d'information patient (FIP, voir l'annexe). Ils auront l'occasion de poser des questions au personnel de l'essai avant de donner leur consentement. Le consentement éclairé, écrit et devant témoin sera enregistré (ICF, voir l'annexe) auprès de chaque participant souhaitant être inclus dans l'essai et d'autres aspects particuliers de l'essai.

L'ICF conçu pour cette étude est basé sur la description contenue dans la dernière version de la déclaration d'Helsinki de 2000 et d'autres documents liés aux BPC. L'approche adoptée pour obtenir le consentement des participants potentiels est décrite dans la section sur l'enrôlement des participants (section 6) du présent protocole.

Le personnel désigné de l'étude examinera le formulaire de consentement avec les éventuels participants qui satisfont les critères d'éligibilité de l'étude. Le formulaire de consentement sera aussi expliqué verbalement dans la langue locale appropriée. Le processus garantira en toute confiance que les éventuels participants comprennent les risques et avantages potentiels, ou le manque d'avantages, du vaccin, les procédures de

CONFIDENTIEL

l'étude, leur droit de refuser et/ou de se retirer de l'étude à tout moment, sans que cela affecte les services ou les soins de santé qu'ils reçoivent, et sans l'obligation d'exposer le motif de leur refus ou de leur sortie de l'étude.

12.6 Vie privée et confidentialité des participants

Le personnel de l'essai devra veiller à ce que l'anonymat des participants soit préservé. Les participants seront uniquement identifiés par les initiales, un numéro d'identification, âge et sexe sur le pCRF et eCRF (d'enrôlement seulement) et ensuite par le numéro d'identification. Tous les documents seront stockés d'une manière sécurisée, et seuls le personnel de l'essai et le personnel autorisé pourront les consulter. L'essai rendra les données anonymes dès qu'il sera possible de le faire sur le plan pratique.

Au début de l'étude, l'investigateur devra préparer un dossier contenant les documents qui se réfèrent à l'essai. Pendant l'étude, l'investigateur sera responsable de la mise à jour du dossier et d'y ajouter régulièrement les documents qui se réfèrent à l'essai. L'investigateur devra conserver le dossier dans une armoire fermée à clé, dans une zone sécurisée à laquelle seuls l'investigateur et le personnel de l'étude peuvent accéder.

Le dossier de l'investigateur et les documents source associés devront être conservés pendant au moins cinq ans après l'achèvement de l'étude. Les codes d'identification des patients devront être conservés pendant au moins 15 ans après l'achèvement de l'étude. Les autorisations écrites de tous les promoteurs devront être obtenues avant de détruire les dossiers.

Tous les documents qui se réfèrent à l'étude devront être conservés dans des armoires fermées à clé sur le site de l'étude. Les armoires de classement contenant les données de l'essai et les informations du participant devront être verrouillées, seules les personnes autorisées par le promoteur et par les autorités de réglementation pourront y accéder.

Les données personnelles du participant resteront confidentielles et la protection des renseignements personnels de tous les participants seront protégés dans la mesure permise par la loi. Les noms des participants apparaîtront sur les documents initiaux et, après le recrutement, les documents de l'étude porteront les initiales des participants et le numéro d'identification attribué. Seul le personnel impliqué dans l'étude et les agences de réglementation locales et internationales peuvent consulter ces dossiers. L'investigateur devra conserver les dossiers de l'investigateur, la liste d'identification des participants et le journal de la sélection / du recrutement (notamment le nom et le prénom, l'âge et l'adresse).

CONFIDENTIEL

13 Aspects administratifs

13.1 Amendements au protocole

Le promoteur modifiera le protocole de l'étude si nécessaire pour faire en sorte que les investigations cliniques soient toujours conduites selon les protocoles appropriés et à jour. Les amendements seront soumis à l'approbation des autorités réglementaires (voir la section 12.3) et les IRB et comités d'éthique (voir la section 12.2). Les modifications nécessitant des modifications seront définies selon les directives des autorités réglementaires et comprennent tout changement significatif affectant la conception de l'étude ou l'ajout d'un nouveau test ou d'une nouvelle procédure.

13.2 Assurance

Le degré de responsabilité du Promoteur est défini par les BPC de l'ICH.

Les participants volontaires impliqués seront assurés, conformément aux lois et réglementations applicables, contre toute perte financière due à des blessures corporelles et / ou autres dommages qui pourraient être une conséquence de cette étude.

Le développement d'un cadre de travail légal pour déterminer un mécanisme de compensation et d'indemnisation qui soit acceptable pour les participants à l'essai en Afrique de l'Ouest, les promoteurs et l'OMS a été initié.

13.3 Financement et autres soutiens pour l'essai

L'essai a été financé par divers organismes et partenaires, notamment l'OMS, l'INSP, le Wellcome Trust, l'ASPC et MSF. Les vaccins seront fournis par le fabricant et un support additionnel sera apporté par les établissements participants.

14 Gestion des données et conservation des dossiers

14.1 Présentation

Un centre de gestion de données (DMC) sera mis en place sur le site de l'étude. La collecte des données aura lieu sur le site de l'étude et sur le terrain en utilisant les formulaires papier standards de Rapport de cas (pCRF). Les pCRF seront transportés par l'équipe désignée de l'étude au Centre de gestion de données. Les commis à la saisie des données utiliseront des ordinateurs portables situés au DMC pour transformer les pCRF en eCRF. La saisie des données sera exécutée par une personne, une autre personne lisant le pCFF et contrôlant la saisie des données en temps réel. Un gestionnaire des données qui rapportera aux investigateurs supervisera les commis à la saisie des données au DMC et gérera la transmission des eCRF à l'équipe de l'analyse statistique. Le centre de gestion des données

sera en communication constante avec le coordonnateur central ou avec les investigateurs principaux locales par le coordinateur de la DAT afin de commenter les problèmes de données (p. ex. questions sur les formulaires papier, questions croisées / modifications du formulaire).

14.2 Dossier du site de l'investigateur (ISF)

Les investigateurs devront conserver les dossiers médicaux et de recherche appropriés pour cet essai, conformément aux BPC E6 de l'ICH et aux exigences réglementaires et institutionnelles pour la protection de la confidentialité des participants. L'investigateur principal, le Co-investigateur, et les infirmières de recherche clinique auront accès aux dossiers. Les investigateurs permettront aux représentants autorisés du (des) promoteur(s), et aux agences de réglementation d'examiner (et si la loi applicable l'exige, de copier) les dossiers cliniques à des fins d'assurance qualité, d'audit et d'évaluation de la tolérance et de la progression de l'étude.

L'ISF sera conservé sur le site de l'essai et inclura au moins les documents et informations suivants :

- protocole et amendements signés
- un exemplaire du formulaire d’exposé de cas (CRF)
- le formulaire de consentement éclairé actuel et toutes les révisions
- Fiche d'information patient actuelle et toutes les révisions
- toutes autres informations écrites remises à l'équipe de l'étude
- aspects financiers de l'étude
- attestation d'assurance
- tous les accords/contrats signés
- approbations du comité d'éthique et des autorités de réglementation datées et documentées
- CV signés de tous les investigateurs et de tout le personnel de l'étude (mis à jour régulièrement en cas de changements)
- instructions pour la manipulation du produit expérimental et des matériels associés à l'étude
- Contrôle des rapports :
    - rapport de lancement de l'étude
    - lettres de suivi des visites sur le site
    - rapport de clôture de l'étude
- Communications pertinentes :
    - lettres
    - compte-rendu des réunions
    - compte-rendu des conversations téléphoniques
- formulaires de consentement éclairé signés

CONFIDENTIEL

- CRF signés, datés, et remplis
- rapports des EIG
- notification par le promoteur des informations sur la tolérance
- rapports intermédiaires et annuels envoyés au comité d'éthique et aux autorités de réglementation
- journal de sélection des participants
- La liste des codes d'identification des participants (correspondance entre patient et ID anonymisé de l'essai)
- journal de recrutement des participants
- responsabilité du médicament expérimental
- fiche d'autorisation / signature
- enregistrement des échantillons de fluides / tissus corporels conservés
- justificatifs de destruction du médicament expérimental
- rapport de l'étude clinique

14.3 Documents-sources

Dans cet essai, les documents suivants ont été identifiés comme « documents-source »:

1. Fiche d'investigation de cas d'Ebola, OMS
2. Résultats laboratoires Ebola
3. Fiche de recherche des contacts Ebola, OMS
4. Formulaire pour le recensement des contacts des cas de la MVE La liste linéaire), OMS
5. Formulaire de suivi des contacts, OMS
6. Formulaire d'exposé de cas (CRFs)

La définition et gestion des documents-sources sont détaillées dans la POS 25.

Le recueil de données avec CRFs sont décrits dans les POS 16.

Si les informations sont collectées à partir de sources différentes que celles spécifiées plus haut, il sera nécessaire de l'enregistrer et de le documenter.

Toutes les procédures requises par protocole ainsi que les informations nécessaires pour rapporter les observations et les tests décrits dans le présent protocole sont enregistrées dans les formulaires papier de rapport de cas (pCRF). En ce qui concerne les entrées rapportées sur les CRF qui proviennent de documents source, p. ex. les résultats des examens de laboratoire, ces documents source seront joints aux dossiers des participants. Si les documents source pour des entrées spécifiques ne sont pas disponibles, cela devra être explicitement mentionné dans le protocole. Pour toutes les informations exigées qui n'ont

CONFIDENTIEL

pas été obtenues comme stipulé dans le protocole, l'explication de ce non obtention devra être notée dans le pCRF. Un personnel indépendant contrôlera la progression de la saisie des données au DMC afin de garantir une collecte opportune et correcte des données.

Les pCRF devront être remplis, signés et autorités de façon opportune et précise par le personnel désigné du site de l'étude dans un délai d'une semaine après la visite. Toutes les données inscrites sur les CRF devront être lisibles, écrites à l'encre bleue ou noire, ou dactylographiées. Tous les CRF seront entrés dans le système REDCap. L'investigateur, ou une personne qualifiée désignée, devra contrôler tous les CRF électroniques (eCRF) à des fins d'exactitude et de cohérence par rapport à la documentation source, puis signer les CRF à l'achèvement du contrôle. Toutes les corrections seront effectuées sur les pCRF en barrant l'entrée incorrecte d'une ligne unique et en saisissant les informations correctes en regard de l'entrée en question. L'investigateur, ou une personne qualifiée désignée, devra dater et parapher la correction. Les pCRF devront ensuite être mise à jour dans le système REDCap avec la documentation appropriée dans l'eCRF pour l'audit des données.

14.4 Tenue des archives

L'ISF, notamment un exemplaire des formulaires d'exposé de cas remplies définitives, ainsi que toute la documentation source, devront être conservés par l'investigateur (et une copie conservés par le promoteur) qui veillera à ce qu'ils soient archivés avec les autres documents de l'étude, comme les formulaires de consentement éclairé signés, le protocole, la brochure de l'investigateur et tous les amendements au protocole, en lieu sûr pendant dix ans.

14.5 Gestion des données

14.5.1 **Matériel et logiciel**

Tous les CRF de cet essai seront entrés dans le CDMS, REDCap. Le système REDCap utilise une base de données MySQL au moyen d'une interface Web sécurisée, avec contrôle des données pendant la saisie afin de garantir la qualité des données. Il comporte également une suite complète de fonctionnalités permettant de prendre en charge la conformité HIPAA, notamment une piste d'audit complète et des privilèges spécifiques aux utilisateurs, et il sera conforme aux exigences des BPC de l'ICH. Le système REDCap ne sera activé pour l'essai qu'après avoir accompli avec succès une procédure formelle de conception et de test. La principale base de données de l'étude sera hébergée sur le serveur REDCap au Centre DMC jusqu'à la clôture formelle de la base de données de l'essai, après quoi la base de données sera transférée à une unité désigné en Europe. Cette unité sera responsable de la mise en œuvre du système REDCap. Le gestionnaire des données sur le site de l'étude sera responsable de la gestion et de la maintenance du serveur REDCap.

La saisie des données des formulaires papier sera effectuée sur des ordinateurs portables. Il est interdit d'utiliser ces ordinateurs portables à des fins autres que les activités de l'étude.

Les ordinateurs portables résideront également sur le site de l'étude et seront connectés au serveur REDCap au moyen d'un réseau local, ce qui n'exige pas de connexion à l'Internet. L'unité désignée en Europe sera responsable de l'achat et de la fourniture des ordinateurs portables. Le gestionnaire des données sur le site de l'étude sera responsable de la gestion et de la maintenance des ordinateurs portables.

14.5.2 **Sécurité, accès et sauvegarde des données**

Le serveur REDCap sera physiquement installé à l'unité désignée en Europe, dans une salle de serveurs verrouillée. Seuls les administrateurs du système ont un accès direct au serveur et aux bandes de sauvegarde. Un concept de rôle avec mots de passe personnels (investigateur du site, statisticien, contrôleur, administrateur, etc.) réglemente les autorisations de chaque utilisateur pour accès au système et à la base de données selon leurs besoins.

Toutes les données saisies dans les CRF sont transférées à la base de données à l'aide d'un codage Secure Sockets Layer (SSL). Des attributs sont attachés à chaque point de données, ce qui permet d'identifier l'utilisateur qui a saisi les données, avec l'heure et la date exactes. Les modifications rétrospectives des données dans la base de données sont enregistrées dans un tableau d'audit. L'heure, le tableau, le champ de données, la valeur modifiée et la personne sont enregistrées (piste d'audit).

Un système de sauvegarde à niveaux multiples est mis en œuvre. Les sauvegardes de tout le système, notamment de la base de données, sont exécutées en interne plusieurs fois par jour, et sur des bandes externes une fois par jour. Les bandes de sauvegarde sont conservées en lieu sûr dans un autre bâtiment.

14.5.3 **Analyses et archivage**

Pour les analyses intermédiaires (si applicable) et l'analyse finale, les fichiers de données seront extraits de la base de données et déposés dans un programme statistique pour analyse. Le statut de la base de données à ce moment-là est enregistré dans des tableaux d'archivage spéciaux.

Le promoteur conservera de manière sécurisée la base de données de l'étude finale et tous les tableaux d'archivage pendant au moins 15 ans. Le promoteur également conservera le dossier permanent de l'essai et les rapports finaux, au format électronique et papier, pendant au moins 10 ans.

15 Assurance et contrôle qualité

15.1 Validation des données électroniques et centrales

La collecte et la saisie des données devront être régulièrement supervisées par une personne désignée au DMC. Cette surveillance centrale sera être prise en charge par l'équipe de gestion des données de l’unité désignée en Europe. Ce processus sera défini dans la POS Gestion des données.

15.2 Contrôle sur le site

15.2.1 **Cadre de monitorage**

L'essai sera mené conformément au protocole actuel approuvé, aux BPC de l'ICH, aux réglementations et procédures d'opérations normalisées pertinentes.

Un suivi régulier sera exécuté selon les BPC de l'ICH, notamment par une visite avant le début de l'essai, des visites régulières dans le cours de l'étude clinique et une visite finale. Les données seront évaluées à des fins de conformité avec le protocole et d'exactitude par rapport aux documents source. Conformément aux procédures d'opérations normalisées, les contrôleurs devront vérifier que l'essai clinique est mené, et les données générées, documentées et rapportées, conformément au protocole, aux BPC et aux exigences réglementaires applicables.

15.2.2 **Mise en œuvre des contrôles et des audits sur les sites de l'étude**

Une société de recherche contractuelle indépendante au Sénégal et le Centre pour le développement des vaccins (CVD) au Mali, va mettre en œuvre le contrôle de l’étude. Les contrôleurs vont participer à toutes les activités importantes qualifiées, en collaboration avec la mise en œuvre de l'étude, qui devront par conséquent être conformes à toutes les visites de contrôle planifiées. Des visites de contrôle seront planifiées à chaque étape de l'étude clinique.

Le programme de surveillance et de vérification sur les sites d'investigation et la fréquence de mise en œuvre du programme de surveillance sont détaillés dans la POS Surveillance de l'étude qui sera rédigée avant l'essai. Les POS couvriront les instructions pour le contrôle des principaux aspects de la mise en œuvre des études cliniques, comme suit :

- autorisations et approbations de l'étude, et communication avec les comités d'éthique
- devoirs et responsabilités de l'investigateur / institution
- ressources appropriées
- soins médicaux pour les participants
- conformité de l'étude clinique avec le protocole convenu

- examens de laboratoire et manipulation des prélèvements
- consentement éclairé des participants
- gestion des données, enregistrements et rapports
- règle d'interruption, arrêt précoce, ou suspension de l'étude
- responsabilités du promoteur
- responsabilités des moniteurs
- responsabilités de l'auditeur

Le stockage et l'intégrité de la chaîne du froid du vaccin expérimental proposé devront être vérifiés afin de garantir que cela est effectué correctement, et à la température indiquée.

En plus des moniteurs, le promoteur va engager un auditeur indépendant pour mener les visites d'audit et pour documenter la conformité avec la BPC.

15.2.3 **Formation pour assurer la qualité de l'essai**

Afin d'assurer le progrès de l'étude et avant le recrutement des éventuels participants, les visites de coordination devront être effectuées avec la direction de l'équipe de recherche clinique sur le site de l'étude, pendant lesquelles la justification et les objectifs de seront expliqués

Une formation sur les BPC sera donnée à l'équipe de l'étude clinique d'essai en utilisant pour cela les POS spécifiques du protocole. L'objectif sera de s'assurer que l'équipe d'étude clinique de l'essai soit bien préparée et entraînée et qu'elle comprenne les détails de l'étude, sa conception et la stratégie consistant à administrer le vaccin proposé sous étude, avant la mise en œuvre de l'étude clinique.

Un atelier d'unification des critères qui formera et vérifiera l'expérience de l'investigateur principal, et du personnel médical, infirmier aura lieu.

15.3 Comité de suivi des données et de la sécurité

Le comité CSDS effectuera un examen par groupe en aveugle et en temps réel des EIG et des données relatives à l'efficacité de l'essai. Les objectifs de ce comité incluent la détection des signaux des EIG, tels que l'augmentation des événements (im) prévus, et la mise en place d'actions appropriées ; la recherche de conseils ou informations supplémentaires auprès des investigateurs au besoin ; l'évaluation du risque de la poursuite de l'essai et la mise en place des mesures appropriées si nécessaire. Le CSDS passera également en revue les données relatives à l'efficacité s'accumulant afin d'établir si et quand l'essai doit être interrompu en raison de sa futilité ou de bénéfices plus importants que prévu. Le CSDS peut souhaiter examiner toutes les données d'autres études de phase I et II des vaccins proposés contre le virus Ebola. Une première réunion du CSDS sera convenue avant le début de l'étude, afin d'examiner le protocole, les rôles et responsabilités, les directives opérationnelles, le plan de

CONFIDENTIEL

contrôle et d'audit. Le CSDS se réunira ensuite régulièrement pendant la durée de l'étude. La mission et fonctions du CSDS seront décrites dans la charte du CSDS.

15.4 Inspections

Les investigateurs et institutions impliqués dans l'étude devront veiller à ce que autorités de réglementation puissent effectuer les contrôles, audits et inspections relativement à la mise en œuvre de l'étude. Cela comprend les examens officiels des documents, des installations, des dossiers, et de toutes les autres ressources que les autorités de réglementation jugent être associées à l'essai clinique et susceptibles de se trouver sur le site de l'essai ou dans les locaux du promoteur et / ou des organismes de recherche de contact, ou dans tout autre établissement jugé approprié par les autorités de réglementation.

15.5 Clôture ou achèvement de l'étude

À conclusion de l'essai, une synthèse des résultats de l'essai devra être remise à l'IRB / IEC sur demande, avec tous les rapports requis, le cas échéant, et tous les rapports devront être transmis aux autorités de réglementation si applicable.

Après clôture ou achèvement de l'étude, tous les documents énumérés dans la section 14.2, ainsi que les allocations d'intervention et le décodage des informations, synthèses et rapports pour l'IRB / IEC ou pour les autorités de réglementation devront être conservés dans le dossier du site de l'investigateur (ISF).

15.6 Politique de diffusion

Le protocole, le jeu de données, et le code statistique seront disponibles sur Internet après que les résultats de l'étude auront été publiés. Une version abrégée du protocole sera publiée dans un journal académique.

15.7 Politique de publication et rapport final

Les résultats de l'étude seront présentés lors de conférences en Guinée, et dans chacun des pays partenaires, ainsi que lors de conférences internationales; le budget de l'étude permettra aux partenaires des pays à revenu faible et à revenu intermédiaire de présenter aux conférences internationales. Les rapports de progression rédigés en temps opportun devront être disponibles pour le MSPH, les donateurs, les responsables de la santé et les autres parties prenantes, notamment les communautés. Le projet publiera les résultats dans des revues scientifiques internationales analysées par des pairs, ainsi que dans les journaux et médias nationaux pertinents. Tous les investigateurs vont être nommés dans les publications. L'équipe de communication de l'OMS facilitera la publication des résultats aux décisionnaires des politiques de santé publique des pays concernés et à l'échelle mondiale.

CONFIDENTIEL

L'impact potentiel de l'étude est la démonstration de l'innocuité et l'efficacité d‘un vaccin contre la MVE dans un contexte d'épidémie à virus Ebola. Les principaux décisionnaires au niveau du pays et de l'OMS seront impliqués dans la consultation en cours et les rapports de progression.

Un rapport final de l'étude sera préparé et transmis aux partenaires concernés, comme décrit ci-dessus.

L’OMS a établi un accord de collaboration avec le fabricant du vaccin et avec d'autres intervenants pour s’assurer que si le vaccin est autorisé pour une utilisation chez l'homme, il sera disponible en quantités suffisantes à un prix abordable pour le secteur de la santé publique en Guinée.

CONFIDENTIEL

16 Références

1 World Health O. Ebola response roadmap - Situation report. http://www.who.int/csr/disease/ebola/situation-reports/en// (accessed Nov 28, 2014).

2 Experimental Ebola vaccines: WHO consultation on Ebola vaccines. Geneva: World Health Organization, October 1, 2014. http://who.int/mediacentre/news/ebola/01-october-2014/en/ (accessed Nov 9, 2014).

3 Kanapathipillai R, Restrepo AMH, Fast P, *et al.* Ebola Vaccine - An Urgent International Priority. *N Engl J Med* 2014; published online Oct 7. DOI:10.1056/NEJMp1412166.

4 Blumberg L, Enria D, Bausch DG. Chapter 16. Viral Haemorrhagic Fevers. In: Manson's Tropical Diseases., 23rd edn. Oxford: Elsevier Expert Consult, 2014.

5 Heffernan RT, Pambo B, Hatchett RJ, Leman PA, Swanepoel R, Ryder RW. Low seroprevalence of IgG antibodies to Ebola virus in an epidemic zone: Ogooué-Ivindo region, Northeastern Gabon, 1997. *J Infect Dis* 2005; **191**: 964–8.

6 WHO Ebola Response Team. Ebola Virus Disease in West Africa - The First 9 Months of the Epidemic and Forward Projections. *N Engl J Med* 2014; **371**: 1481–95.

7 Leroy EM, Kumulungui B, Pourrut X, *et al.* Fruit bats as reservoirs of Ebola virus. *Nature* 2005; **438**: 575–6.

8 Leroy EM, Epelboin A, Mondonge V, *et al.* Human Ebola outbreak resulting from direct exposure to fruit bats in Luebo, Democratic Republic of Congo, 2007. *Vector Borne Zoonotic Dis* 2009; **9**: 723–8.

9 Okware SI, Omaswa FG, Zaramba S, *et al.* An outbreak of Ebola in Uganda. *Trop Med Int Health* 2002; **7**: 1068–75.

10 Dowell SF, Mukunu R, Ksiazek TG, Khan AS, Rollin PE, Peters CJ. Transmission of Ebola hemorrhagic fever: a study of risk factors in family members, Kikwit, Democratic Republic of the Congo, 1995. Commission de Lutte contre les Epidémies à Kikwit. *J Infect Dis* 1999; **179 Suppl** : S87–91.

11 Bah EI, Lamah M-C, Fletcher T, *et al.* Clinical Presentation of Patients with Ebola Virus Disease in Conakry, Guinea. *N Engl J Med* 2014; : 141105140010007.

12 Forrester JD, Hunter JC, Pillai SK, *et al.* Cluster of ebola cases among liberian and u.s. Health care workers in an ebola treatment unit and adjacent hospital - liberia, 2014. *MMWR Morb Mortal Wkly Rep* 2014; **63**: 925–9.

CONFIDENTIEL

13 Sullivan NJ, Martin JE, Graham BS, Nabel GJ. Correlates of protective immunity for Ebola vaccines: implications for regulatory approval by the animal rule. *Nat Rev Microbiol* 2009; **7**: 393–400.

14 OMS. Ebola Response Roadmap Situation Report. 15 janvier 2015 .

15 Organization WH. Clinical Management of Patients with Viral Haemorrhagic Fever: A Pocket Guide for the Front-line Health Worker. Geneva, 2014.

16 Organization WH. Infection prevention and control guidance for care of patients in health-care settings, with focus on Ebola. http://www.who.int/csr/resources/publications/ebola/filovirus_infection_control/en/ (accessed Dec 22, 2014).

17 Organization WH. How to safely collect blood samples from persons suspected to be infected with highly infectious blood-borne pathogens (e.g. Ebola). http://www.who.int/csr/resources/publications/ebola/blood-collect/en/ (accessed Dec 22, 2014).

18 Organization WH. Personal protective equipment in the context of Filovirus disease outbreak response. http://www.who.int/csr/resources/publications/ebola/ppe-guideline/en/ (accessed Dec 22, 2014).

19 Organization WHRO for A. Contact Tracing During an Outbreak of Ebola Virus Disease. Brazzaville, 2014 http://www.who.int/csr/resources/publications/ebola/contact-tracing-during-outbreak-of-ebola.pdf (accessed Nov 29, 2014).

20 Roberts I, Perner A. Ebola virus disease: clinical care and patient-centred research. *Lancet* 2014; **384**: 2001–2.

21 Althaus CL. Estimating the Reproduction Number of Ebola Virus (EBOV) During the 2014 Outbreak in West Africa – PLOS Currents Outbreaks. *PLoS Curr Outbreaks* 2014; **Sep 2. Edi**: 1–9.

22 Henderson DA, Klepac P. Lessons from the eradication of smallpox: an interview with D. A. Henderson. *Philos Trans R Soc Lond B Biol Sci* 2013; **368**: 20130113.

23 Kretzschmar M, van den Hof S, Wallinga J, van Wijngaarden J. Ring vaccination and smallpox control. *Emerg Infect Dis* 2004; **10**: 832–41.

24 Ledgerwood JE, DeZure AD, Stanley DA, *et al.* Chimpanzee Adenovirus Vector Ebola Vaccine — Preliminary Report. *N Engl J Med* 2014; : 141126135947008.

25 Bonhoeffer J, Bentsi-Enchill A, Chen RT, *et al.* Guidelines for collection, analysis and presentation of vaccine safety data in pre- and post-licensure clinical studies. *Vaccine* 2009; **27**: 2282–8.

CONFIDENTIEL

26 Donner A, Klar N. Statistical considerations in the design and analysis of community intervention trials. *J Clin Epidemiol* 1996; **49**: 435–9.

27 Donner A, Klar N. Design and Analysis of Cluster Randomization Trials in Health Research. London: Arnold, 2000.

28 Response UM for EE. Ebola Virus Disease Outbreak Response Plan in West Africa. http://reliefweb.int/report/guinea/ebola-virus-disease-outbreak-response-plan-west-africa (accessed Nov 29, 2014).

29 (UNAIDS) JUNP on H. Good participatory practice. Guidelines for biomedical HIV prevention trials 2011. Geneva, 2011.

30 Executive H and S. The Genetically Modified Organisms (Contained Use) Regulations 2014. Guidance on regulations L29 - l29.pdf. London.

31 World Medical A. WMA Declaration of Helsinki - Ethical Principles for Medical Research Involving Human Subjects. Fortaleza, 2013.

32 Geisbert TW, Feldmann H. Recombinant vesicular stomatitis virus-based vaccines against Ebola and Marburg virus infections. *J Infect Dis* 2011; **204 Suppl** : S1075–81.

33 Hoenen T, Groseth A, Feldmann H. Current ebola vaccines. *Expert Opin Biol Ther* 2012; **12**: 859–72.

34 Geisbert TW, Geisbert JB, Leung A, *et al.* Single-injection vaccine protects nonhuman primates against infection with marburg virus and three species of ebola virus. *J Virol* 2009; **83**: 7296–304.

35 Stanley DA, Honko AN, Asiedu C, *et al.* Chimpanzee adenovirus vaccine generates acute and durable protective immunity against ebolavirus challenge. *Nat Med* 2014; **20**: 1126–9.

36 Swiss trial of Canadian Ebola vaccine halted temporarily - The Globe and Mail. http://www.theglobeandmail.com/news/national/swiss-trial-of-canadian-ebola-vaccine-halted-temporarily/article22047697/ (accessed Dec 21, 2014).

37 World Health O. Laboratory Guidance for the Diagnosis of Ebola Virus Disease. Interim Recommendations. 19 September 2014. http://apps.who.int/iris/bitstream/10665/134009/1/WHO_MVE_GUIDANCE_LAB_14.1_eng.pdf?ua=1 (accessed Dec 22, 2014).

38 Aalen O, Borgan O, Gjessing H. Survival and Event History Analysis - A Process Point of View. 2008.

CONFIDENTIEL

39 Munda M, Rotolo F, Legrand C. parfm: Parametric Frailty Models in R. *J Stat Softw* 2012; **51**: 1–20.

40 Good Clinical Practice (CPMP/ICH/135/95). http://ec.europa.eu/health/files/eudralex/vol-10/3cc1aen_en.pdf (accessed Dec 22, 2014).

41 Organization WH. Meeting of the Ethics Working Group on Ebola Interventions. Geneva, Switzerland 20 – 21 October 2014. http://www.who.int/medicines/wg_ethics_ebola_interventions/en/ (accessed Dec 22, 2014).

42 Recommendations from the 9th Annual Meeting of the African Vaccine Regulatory Forum (AVAREF) Pretoria, 3 to 7 November 2014. http://www.who.int/immunization_standards/vaccine_regulation/avaref_meeting_recommendations_14nov2014.pdf?ua=1 (accessed Dec 22, 2014).

43 FDA. CFR - Code of Federal Regulations Title 21. http://www.accessdata.fda.gov/scripts/cdrh/cfdocs/cfcfr/CFRSearch.cfm?fr=312.30 (accessed Dec 22, 2014).

17 Annexes

CONFIDENTIEL
